# Spatial Distribution and Stability of Cholinesterase Inhibitory Protoberberine Alkaloids from *Papaver setiferum*

**DOI:** 10.1021/acs.jnatprod.1c00980

**Published:** 2021-12-15

**Authors:** Neda Safa, Tomaž Trobec, Darren C. Holland, Blazej Slazak, Erik Jacobsson, Jeffrey A. Hawkes, Robert Frangež, Kristina Sepčić, Ulf Göransson, Lindon W. K. Moodie, Luke P. Robertson

**Affiliations:** † Pharmacognosy, Department of Pharmaceutical Biosciences, Biomedical Centre, Uppsala University, 75237 Uppsala, Sweden; ‡ Drug Design and Discovery, Department of Medicinal Chemistry, Biomedical Centre, Uppsala University, 75123 Uppsala, Sweden; § Institute of Preclinical Sciences, Veterinary Faculty, University of Ljubljana, 1000 Ljubljana, Slovenia; ∧ School of Environment and Science, Griffith University, Southport 4222, Gold Coast, Australia; ∥ Griffith Institute for Drug Discovery, Griffith University, 4111 Nathan, Australia; ∇ W. Szafer Institute of Botany, Polish Academy of Science, 46 Lubicz Street, 31-512, Kraków, Poland; □ Analytical Chemistry, Department of Chemistry, Biomedical Centre, Uppsala University, 75120 Uppsala, Sweden; # Department of Biology, Biotechnical Faculty, University of Ljubljana, 1000 Ljubljana, Slovenia; ~ Uppsala Antibiotic Centre, Biomedical Centre, Uppsala University, 75123 Uppsala, Sweden

## Abstract

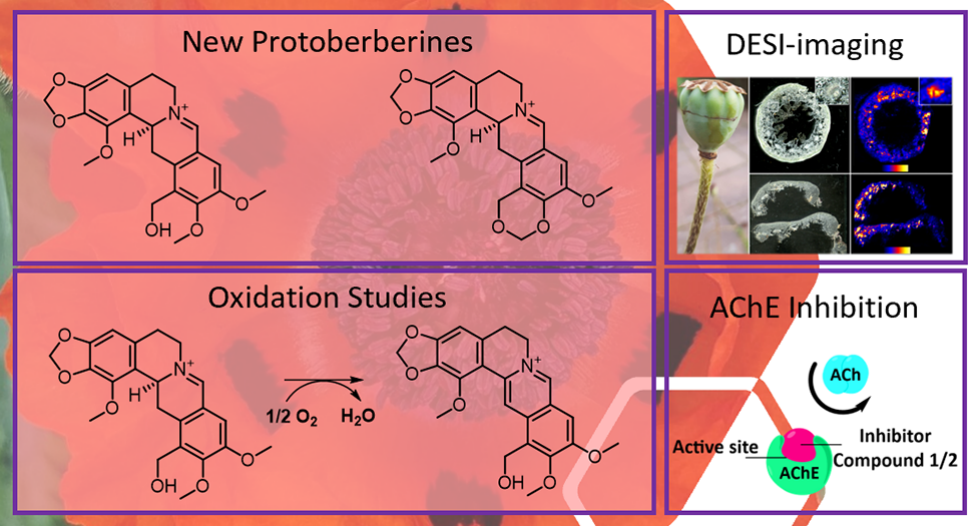

During a research program to identify new cholinesterase inhibitors of natural origin, two new 7,8-didehydroprotoberberine alkaloids (**1** and **2**) and nine known compounds (**3**–**11**) were isolated from the capsules of the common ornamental poppy, *Papaver setiferum* (previously *P. pseudo-orientale*). Despite their reported instability, the 7,8-didehydroprotoberberines isolated herein appeared relatively stable, particularly as their trifluoroacetic acid salts. The spatial distributions of the isolated alkaloids were also analyzed using desorption electrospray ionization imaging mass spectrometry. The alkaloids were localized predominantly within the walls and vascular bundles of the capsules, with the highest relative abundances occurring in the lower half of the capsules toward the peduncle. The relative abundances of the alkaloids were also compared across plant development stages. Although most alkaloids did not show clear patterns in their concentration across development stages, the concentration of suspected oxidation products clearly spiked upon plant death. Finally, all isolated natural products were screened for inhibitory activities against a panel of cholinesterases, from both human and animal sources. These studies identified several competitive inhibitors of cholinesterases with potency in the low micromolar range (**1**–**4**, **6**, **7**), offering new lead compounds for the development of cholinesterase inhibitory drugs.

Cholinesterases are a family of enzymes consisting of two main classes. Acetylcholinesterase (AChE, E.C. 3.1.1.7, or “true cholinesterase”) is a serine hydrolase that catalyzes the hydrolysis of the neurotransmitter acetylcholine. In mammals, AChE is present in cholinergic synapses of the central, peripheral, and autonomic nervous systems and in neuromuscular junctions and erythrocytes.^1−3^ Butyrylcholinesterase (BChE, E.C. 3.1.1.8, previously called pseudocholinesterase) is a nonspecific cholinesterase that hydrolyzes a variety of choline and noncholine esters and is found primarily in plasma and in the liver.^1,4^ Both cholinesterases can catalyze the hydrolysis of acetylcholine to terminate neurotransmitter activity and signal transduction.^3−6^

Drugs that inhibit the activity of cholinesterases have emerged as leading symptomatic treatments for the early stages of Alzheimer’s disease to date.^7^ In the early stages of Alzheimer’s disease, patients have enhanced AChE activity, resulting in decreased acetylcholine levels in the brain.^8^ The results of some studies show that with the progression of Alzheimer’s disease the expression of AChE decreases, while the expression of BChE begins to increase in specific brain areas. Drugs with cholinesterase inhibitory activity prolong the lifetime of acetylcholine, resulting in improvement of cholinergic function and alleviation of symptoms.^6,9−11^ AChE inhibitors are also used in the treatment of myasthenia gravis, a long-term neuromuscular disease.^1,8^ However, these drugs are not without negative side effects, and there is an ongoing need for the development and discovery of new and improved treatments.

In both cholinesterases, acetycholine hydrolysis occurs at the bottom of a deep “gorge” that is located ca. 2 nm from the enzyme surface. The bottom of this gorge contains both anionic and esteratic sites. The anionic site binds to the quaternary ammonium ion of acetylcholine through cation−π interactions, while the esteratic site is responsible for ester hydrolysis.^12^ Competitive binding to the anionic site of AChE is a common mechanism for AChE inhibitors, thereby limiting access of the substrate to the catalytic site. These inhibitors thus often contain quaternary ammonium or tertiary amine groups that mimic the ammonium group of acetylcholine.^8,12^ Alkaloids from plants in particular have been a prominent source of cholinesterase inhibitors, such as the clinically used drugs galantamine and physostigmine.^13^

The *Papaver* genus (Papaveraceae) is best known as the source of morphine and codeine.^14^ Other than these, *Papaver* is a prolific producer of other alkaloids, with more than 200 examples reported from the genus in the *Dictionary of Natural Products*.^15^ To expand on previous work on natural cholinesterase inhibitors,^16,17^ the chemical constituents of the common ornamental poppy, *Papaver setiferum* Goldblatt, were studied.^18^*P. setiferum* was known as *P. pseudo-orientale* (Fedde) Medw. from 1918 until it was changed to *Papaver setiferum* Goldblatt in 2011.^19^ A proposal to conserve the name *P. pseudo-orientale* (Fedde) Medw. was recently published,^20^ although this is yet to be voted on by the Nomenclatural Committee for Vascular Plants.^21^

It can be assumed that defense compounds are stored within plant tissues in regions that are associated with their biological activities; for example, antimicrobial and insecticidal compounds may be stored in the epidermis. Therefore, compound distributions within plant organs may predict their biological functions.^22,23^ The recent introduction of mass spectrometry imaging techniques with ambient ionization has made it significantly easier to study natural product distribution within plant organs and tissues. The newly emerging technique desorption electrospray ionization imaging mass spectrometry (DESI-IMS) offers multiple advantages over the more well-established matrix-assisted imaging mass spectrometry (MALDI-IMS). These include simplified sample preparation, less interference in the low mass range (<300 *m*/*z*), and fewer artificially introduced matrix effects.^24^ To the best of our knowledge, there have been no studies utilizing direct DESI-IMS to depict the distribution of alkaloids in *Papaver* tissues so far.

Described herein is the isolation, identification, in situ localization, and cholinesterase inhibitory screening of two new and nine known compounds from the capsules of *P. setiferum*.

## Results and Discussion

### Isolation and Structural Elucidation of *Papaver* Alkaloids

**Figure 1:**
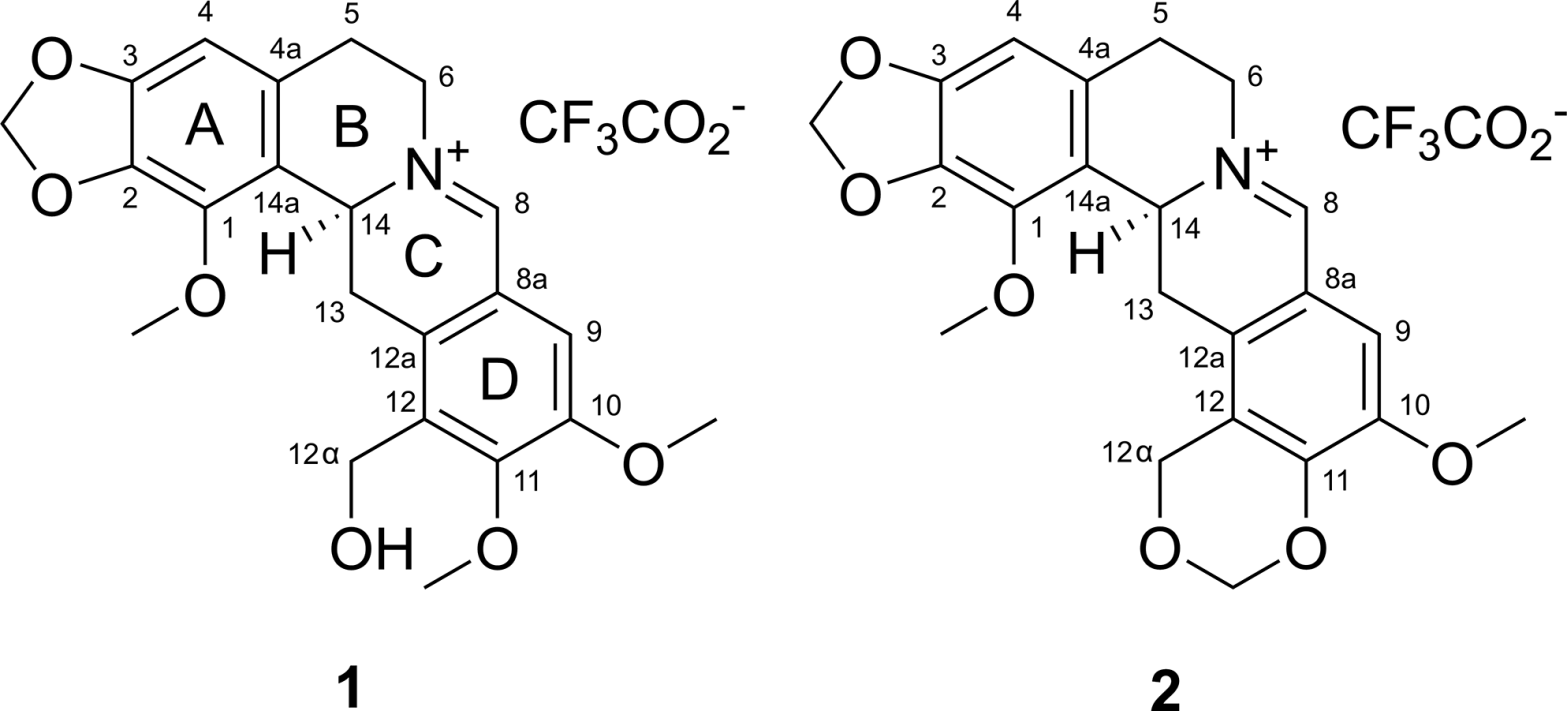
New 7,8-didehydroprotoberberine alkaloids isolated from *P. setiferum*: 7,8-didehydromecambridine TFA salt (**1**) and 7,8-didehydroorientalidine TFA salt (**2**).

Extraction of dried *P. setiferum* capsules with MeOH/CH_2_Cl_2_ followed by reversed-phase HPLC led to the isolation of two new protoberberine alkaloids as their TFA salts (**1** and **2**) (Figure 1). Eight previously described alkaloids (**3**–**10**) and a nitroethyl-containing phenolic glycoside (**11**) were also isolated.

7,8-Didehydromecambridine TFA salt (**1**) was isolated as a yellow amorphous solid. HRESIMS in the positive mode showed a prominent molecular ion peak at *m*/*z* 398.1597 [M – TFA]^+^, corresponding to a molecular formula of C_22_H_24_NO_6_^+^ (calcd 398.1598). Analysis of its ^1^H and HSQC NMR data (Table 1) showed that **1** contains two aromatic singlets (δ_H_ 7.60, 6.69, 2H), five methylene groups (δ_H_ 6.07–2.78, 10H), one aliphatic methine (δ_H_ 5.27, 1H), and three aromatic methoxy groups (δ_H_ 4.04, 3.90 3.90, 9H). A deshielded methine (δ_H_ 9.29, 1H; δ_C_ 165.7) was attributed to an iminium group based on both its chemical shift and ^1^*J*_CH_ coupling (190 Hz) as observed from an F2-coupled HSQC experiment (Supporting Information, Figure S7).^25,26^ Twelve additional sp^2^-hybridized carbons were featured in the ^13^C NMR spectrum of **1** (δ_C_ 154.5–103.0), suggesting the presence of two highly substituted benzene rings. Comparison of these data with similar previously isolated alkaloids indicated that **1** contains a 7,8-didehydroprotoberberine backbone, although with different substitution patterns on rings A and D.^25,26^ The lack of any shared HMBC correlations between the aromatic singlets (δ_H_ 7.60, 6.69) indicated that **1** contains two pentasubstituted benzene rings. A resonance characteristic of a methylenedioxyphenyl motif (δ_H_ 6.07/6.02, δ_C_ 101.4) was assigned to ring A based on shared ^3^*J*_CH_ correlations with H-4 (δ_H_ 6.69). A weak ^4^*J*_CH_ correlation from H-4 to C-1 was used to assign the methoxy group at C-1, and a ROESY correlation between H-4 and H-5 confirmed their positions relative to each other.^27,28^ The structure of ring D was determined by ^3^*J*_CH_ HMBC correlations from H-9 to C-11/C-12a and a ROESY correlation between H-9 and MeO-10. Finally, the hydroxymethyl group (C-12α; δ_H_ 4.60/4.54, δ_C_ 53.8) was assigned based on ^2^*J*_CH_ and ^3^*J*_CH_ HMBC correlations to C-11, C-12, and C-12a. HMBC and ROESY correlations supported the assignment of C-9 as beta to the imine carbon and thus *para* to the hydroxymethyl group. After the 2D structure of **1** was established, the absolute configuration of C-14 was determined by comparing experimental and predicted ECD spectra, calculated using time-dependent density functional theory (TDDFT) (Figure 3). Based on these data, the absolute configuration of **1** was assigned as 14*S*.

**Figure 2:**
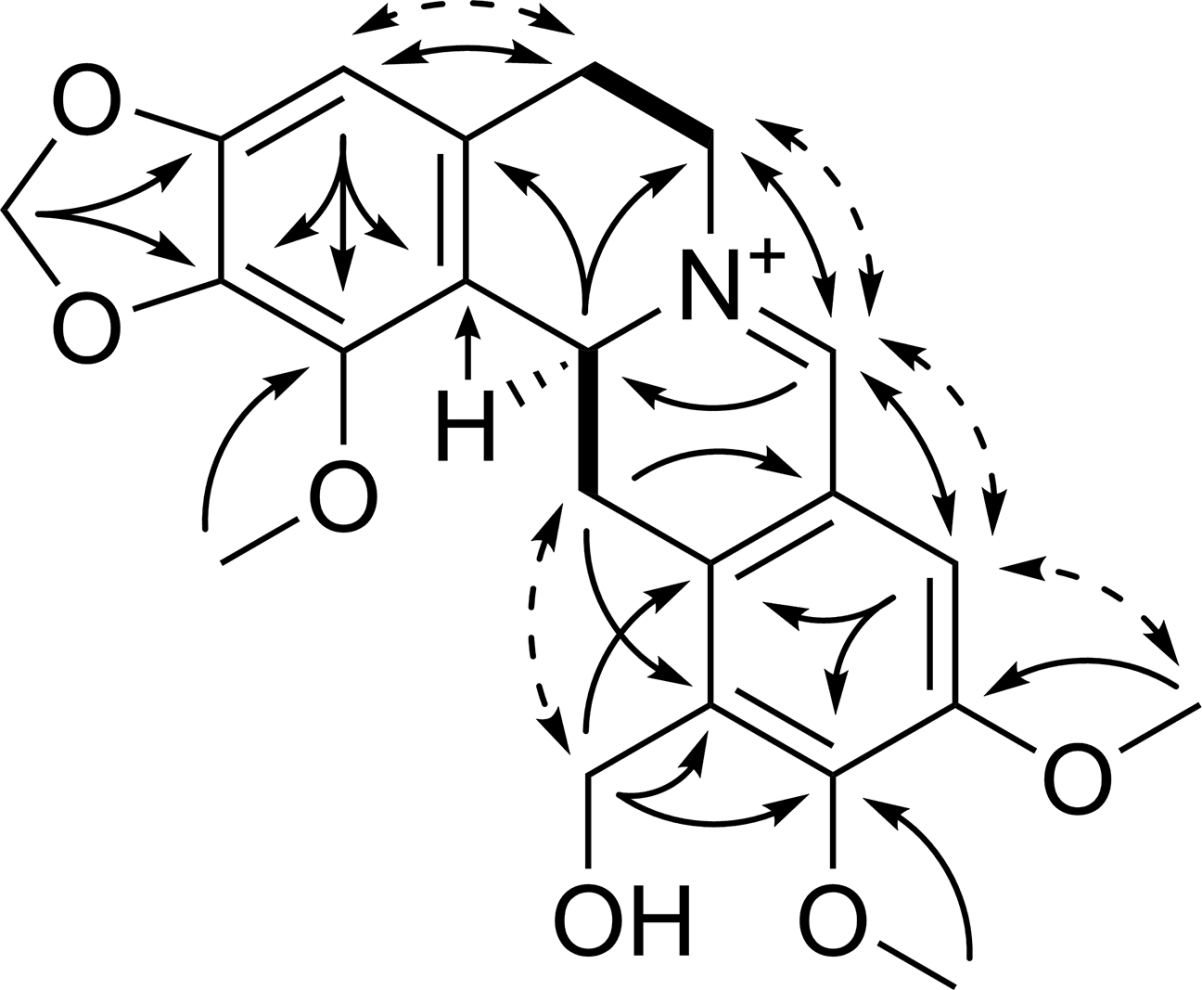
Key HMBC (solid arrows), ROESY (dashed arrows), and COSY (bold bonds) correlations in **1**.

**Figure 3:**
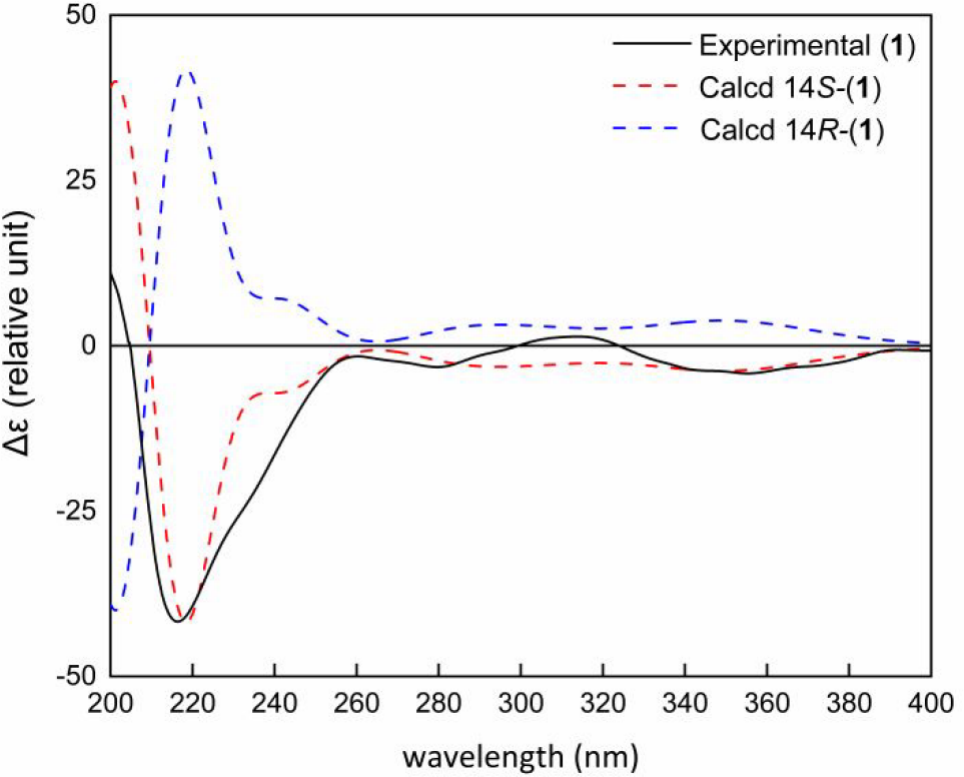
Comparison of experimental (solid black line) with TDDFT-calculated ECD spectra (dashed lines) of **1** at the B3LYP/6-311++G(d,p) level of theory.

7,8-Didehydroorientalidine TFA salt (**2**) was isolated as a yellow amorphous solid. HRESIMS in the positive mode gave a molecular ion peak at *m*/*z* 396.1441 [M – TFA]^+^, corresponding to a molecular formula of C_22_H_22_NO_6_^+^ (calcd 396.1442). Comparison of the molecular formula and NMR spectra of **2** with **1** suggested a high level of structural similarity, where an additional degree of unsaturation in **2** was a notable distinction. The main differences between the ^1^H and HSQC spectra of **1** and **2** included the loss of an aromatic methoxy group in **2**, while the hydroxymethyl group at C-12α in **1** (δ_C_ 53.8, δ_H_ 4.60/4.54) was comparatively deshielded (δ_C_ 63.4, δ_H_ 5.04/4.89). Other than these resonances, comparison of the ^13^C NMR data between **1** and **2** suggested highly similar structures, except for the resonances associated with C-8a, C-9, C-10, C-11, C-12, and C-12a, all of which were observed as slightly shielded in **2**. Further analysis confirmed that the compounds are analogous with the exception that **2** contains a 1,3-dioxane moiety at C-11/C-12. The absolute configuration of **2** (14*S*) was found to be the same as that assigned to **1** by comparison of their experimental ECD spectra and optical rotation values (Supporting Information, Figure S21).

**Figure 4:**
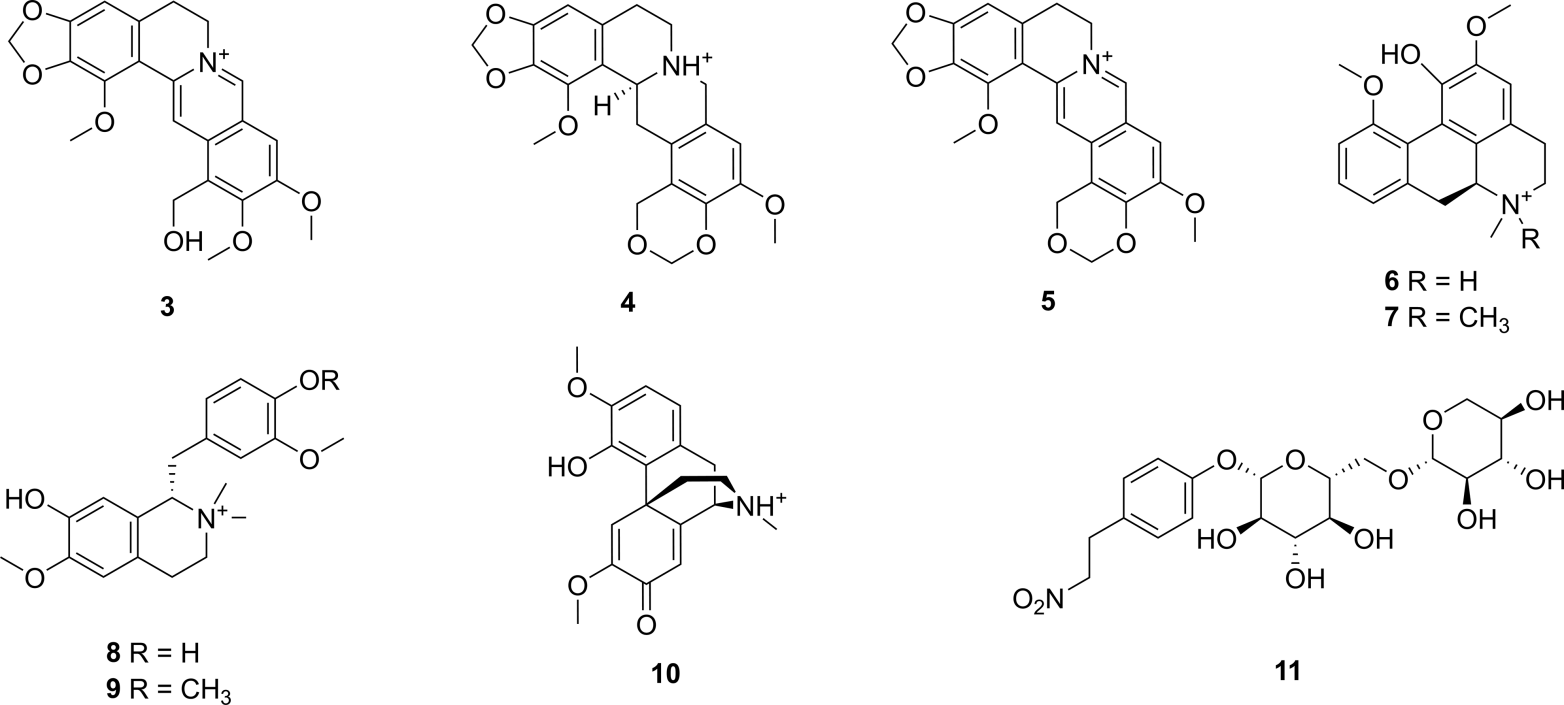
Known compounds (**3**–**11**) isolated from *P. setiferum*.

The known compounds alborine (**3**),^29^ orientalidine (**4**), 7,8,13,14-dehydroorientalidine (**5**), isothebaine (**6**),^30^*N-*methylisothebainium (**7**),^18^*N*-methylorientaline (**8**), *N*-methylcodamine (**9**),^18^ salutaridine (**10**),^31^ and thalictricoside (**11**)^32^ were also isolated from the capsules of *P. setiferum* (Figure 4). The NMR data for **5** have not been previously reported and are provided in the Supporting Information (Table S23). The structures of known compounds were determined by MS and 2D NMR analysis, then confirmed by comparison of NMR data with literature values (data not shown). The absolute configurations for co-isolates containing stereocenters were determined with optical rotatory data.

*P. setiferum* appears to be a particularly rich source of *N*,*N*-dimethylated quaternary ammonium alkaloids (**7**–**9**) when compared with other *Papaver* species. Two of these (**8** and **9**) have only been isolated from *P. setiferum*,^18^ while **7** appears restricted to species within *Papaver* sect. *Macrantha* Elkan.^18,33^ The nitroethyl-containing phenolic glycoside **11** has been found previously in plants from the Menispermaceae,^34^ Ranunculaceae,^32^ and Aristolochiaceae families.^35^ Interestingly, these families all produce strikingly similar alkaloid profiles to *Papaver* in terms of benzylisoquinolines and their downstream biosynthetic products (e.g., aporphines and/or protoberberines).^36,37^ 4-(2-Nitroethyl)phenol, a presumed biosynthetic precursor of **11**, is produced from tyrosine in response to osmotic stress in cell cultures of *Eschscholzia californica* (Papaveraceae). Subsequent observations that 4-(2-nitroethyl)phenol is readily glucosylated establishes a putative biosynthetic pathway toward **11**.^38^ Tyrosine has also been shown as the biosynthetic precursor to 4-(2-nitroethyl)phenol in *Sorghum bicolor* (Poaceae).^39^

### Alkaloid Spatial Mapping

**Figure 5:**
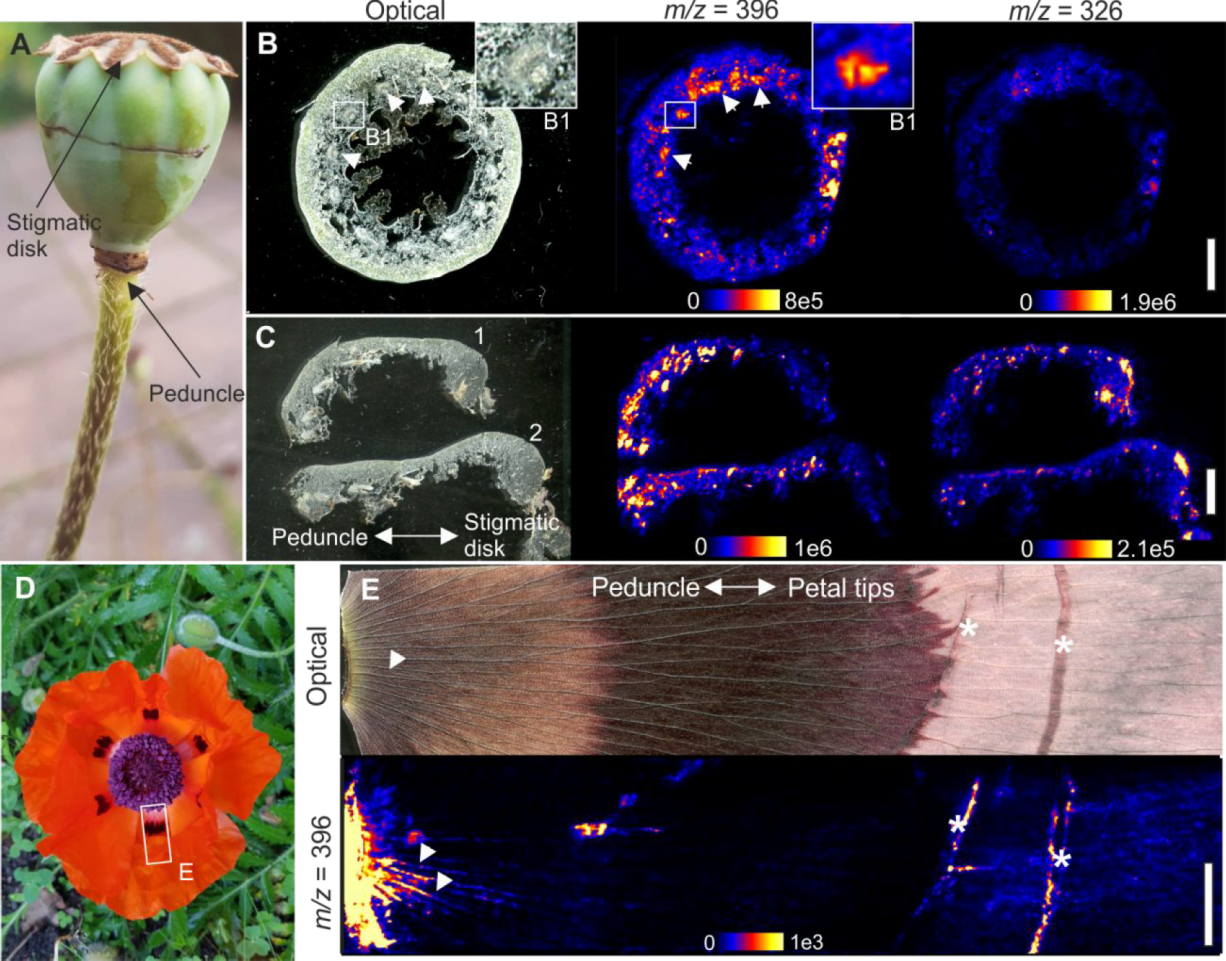
DESI-IMS data of the distribution of compounds **2**/**3** (*m*/*z* 396) and **7** (*m*/*z* 326) in *P. setiferum* capsules and flower petals. (A) Capsule with simple anatomy marked. (B) Horizontal cross-section of the capsule taken from the lower half with alkaloids shown in the pod walls and vascular bundles (marked with arrowheads and magnified in B1). (C) Vertical cross sections through two different capsules (1, 2). (D) *P. setiferum* flower. (E) DESI-IMS data of a flower petal fragment. Vascular tissues are marked with arrowheads. The folds occurring during preparation of the petals for imaging caused the appearance of some imaging artifacts (marked with asterisk). Bar = 3 mm (B); 3.5 mm (C); 4.5 mm (E).

The spatial distributions of the isolated alkaloids within *P. setiferum* were mapped using DESI-IMS. Cross sections of the capsules (Figure 5a) were prepared both along their horizontal (Figure 5b) and vertical (Figure 5c) axes using a cryostat microtome. Imaging data from the horizontal cross-section of the capsules showed that compounds with *m*/*z* ions at 396 (corresponding to **2**/**3**; indistinguishable due to their identical molecular formulas) are accumulated in the capsule walls, with highest relative abundances close to and inside the vascular tissues (Figure 5b). Other isolated alkaloids (**1**, **4**–**6**, **8**–**10**) showed an almost identical pattern of distribution (Supporting Information, Figure S30). Compound **7** (*m*/*z* 326) was the only exception, showing rising relative abundances toward the stigmatic disk (Figure 5c).

Alkaloids are known to protect plants against herbivory, and those of the protoberberine (**1**–**5**), aporphine (**6** and **7**), and benzylisoquinoline (**8** and **9**) types have all been shown to deter insect feeding.^40−42^ Methylenedioxyphenyl-containing compounds (e.g., **1**–**5**) may also improve synergistically the activity of other antifeedants through the inhibition of mixed-function oxidase enzymes responsible for the metabolism of xenobiotics, e.g., cytochrome P450s (see piperonyl butoxide).^42−44^ The distributions and known biological activities of the alkaloids in the capsules shown herein imply that they are produced to provide protection for the developing seeds. The walls of the capsule are the thickest in the nutrient-rich base, where it connects to the peduncle. Presumably, the relative abundances of most alkaloids are the highest here to protect the main source of nourishment for the developing seeds. Other explanations may be that they accumulate in the lower half of the capsule as they are translocated from the stem, or alternatively that their biosynthesis occurs here. The unique distribution of **7** in the two capsules analyzed may reflect specialization of this compound to target a specific herbivore or pathogen that attacks the capsule from the stigmatic disk. However, this pattern would need to be confirmed through additional biological replicates.

Alkaloids were also found in the flower petals, in the vascular tissues, and in the region at the base of the petals, where they attach to the capsule (Figure 5d,e). Most likely, they seep into the petals from the alkaloid-rich tissues through the vasculature. The leaves were also imaged, but no compounds could be detected, likely due to the thickness of the cuticle. To the best of our knowledge, this is the first study analyzing a *Papaver* sp. by direct DESI-IMS. An earlier study reported the use of an indirect DESI-IMS method to analyze *P. somniferum*.^45^ In this investigation, imprints made by pressing sliced capsules against Teflon plates were imaged instead of the specimens directly. The image essentially resulted in a map of the distribution of latex within the capsule, and no data on the longitudinal distribution of the alkaloids were acquired.

### Stability of 7,8-Didehydroprotoberberines

**Figure 6:**
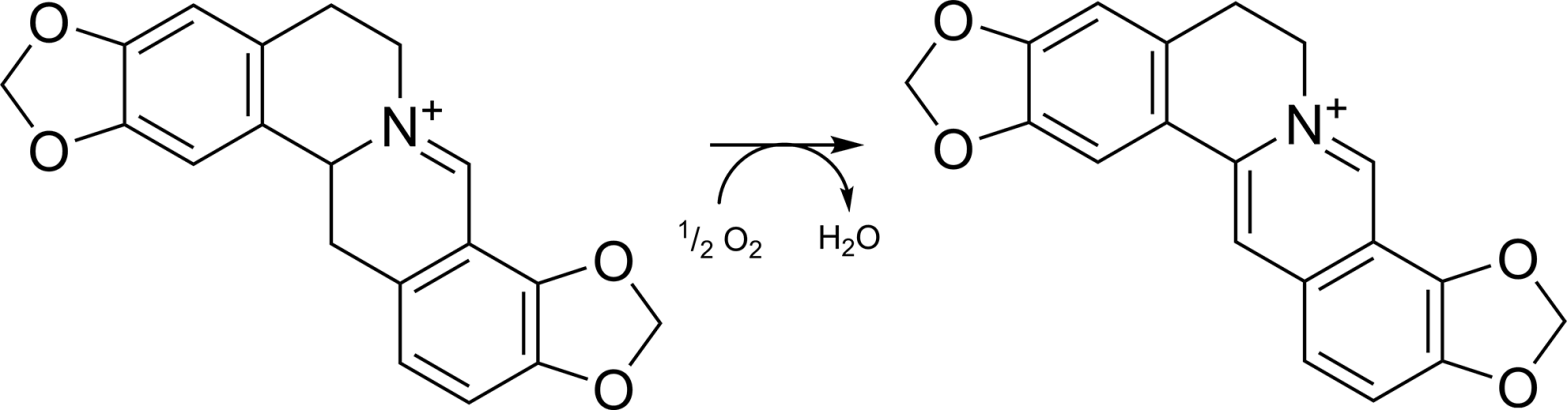
Conversion of 13,14-dihydrocoptisine to coptisine in *C. majus* after tissue injury^26^

Compounds **1** and **2** are the first 7,8-didehydroprotoberberines to be isolated from *Papaver*. To the best of our knowledge, naturally occurring 7,8-didehydroprotoberberines have only been reported previously from the stems of *Annona glabra* (Annonaceae)^25^ and the leaves of *Chelidonium majus* (Papaveraceae).^26^ Their scarcity in the literature may be due to their apparent instability: the challenges faced in the purification of 13,14-dihydrocoptisine from *C. majus* were the primary focus of work on its isolation.^26^ It was found that 13,14-dihydrocoptisine is the “true” naturally occurring alkaloid of *C. majus*, but it is rapidly oxidized to coptisine after tissue injury (Figure 6). Even after a carefully designed extraction procedure (flash-freezing of leaves in liquid nitrogen and direct extraction in MeOH/diluted acetic acid [pH 3.5; 75:25, v/v]), the authors still experienced great difficulty in isolating the compound due to its tendency to oxidize during freeze-drying of HPLC fractions.

These findings are of interest as they contrast with observations from the present work. In this study, no special precautions were taken in the isolation of **1** and **2**, although the laboratory studies were conducted relatively quickly: only 9 days elapsed from the collection of fresh plants to the acquisition of NMR data (see Supporting Information, Figure S31 for a detailed isolation timeline). The compounds later appeared particularly stable as their TFA salts: a dried sample of **1** (0.7 mg) stored in an uncapped glass vial at room temperature for approximately 8 weeks showed no oxidation to **3**. Interestingly, the other study describing naturally occurring 7,8-didehydroprotoberberines also did not report difficulties with their stability.^25^ Comparison of the present methodology with the approaches used by Tsai and Lee^25^ and Paulsen et al.^26^ has led to the hypothesis that the use of a strong acid (0.1% TFA, pH ∼2.0) during HPLC may be an important component of compound stability. In the present investigation, 0.1% TFA was used in both solvents during HPLC purification, and the same was done by Tsai and Lee. Conversely, Paulsen et al. opted for a three-solvent system of 10 mM (NH_4_)_2_SO_3_, 0.2% triethylamine adjusted with acetic acid to pH 4.0 (solvent A), CH_3_CN (solvent B), and MeOH (solvent C). Their observation that 13,14-dihydrocoptisine oxidized repeatedly after HPLC leads to the present conclusion that these conditions may have been insufficiently acidic to stabilize the compound. However, this was not tested in the current work. Further, given the varied substitution of the aromatic motifs in these compounds, the role these groups play upon stability cannot be yet ascertained.

**Figure 7:**
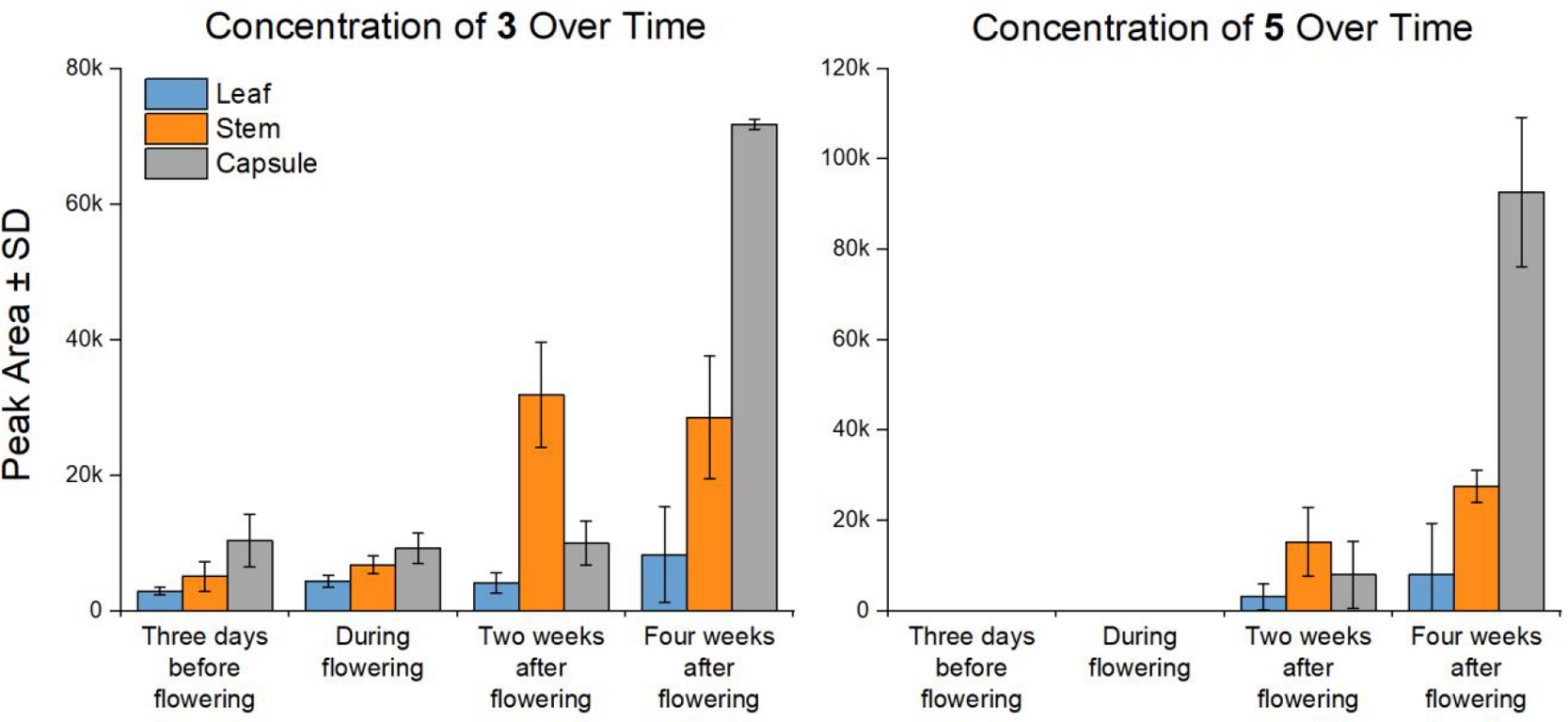
Concentration of **3** and **5** across several flowering stages of *P. setiferum*. Compound **5** was below the limit of detection (LOD) at the first two time points.

**Figure 8:**
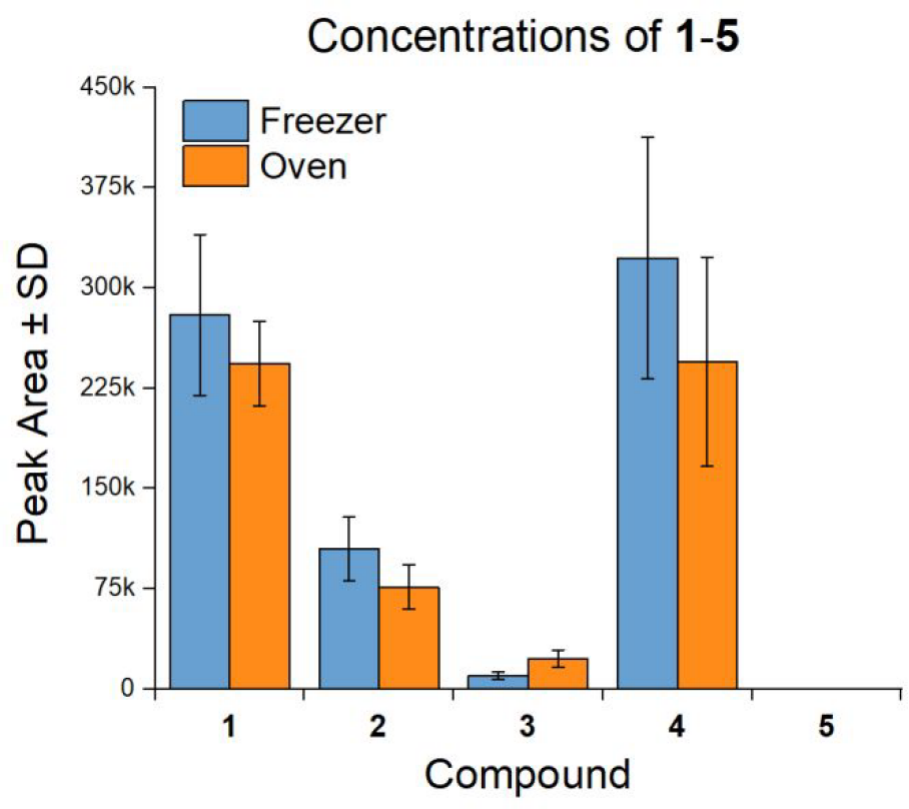
Concentration of **1**–**5** in *P. setiferum* capsules stored in an oven (2 weeks, 40 °C) vs those stored in a freezer (−20 °C). Compound **5** remained below the limit of detection (LOD) in all samples.

To further understand the stability and distribution of the alkaloids, their concentrations were compared in different plant parts at four time points (3 days before flowering, while flowering, 2 weeks after flowering, and 4 weeks after flowering) (Figure 7). Alkaloids were generally present in the highest relative concentrations in the capsules, followed by the stems and then leaves. The concentration of compounds **3** and **5** (the oxidized variants of **1** and **2**, respectively) dramatically increased in all aerial parts as the plant dried and began to die at the four-week time point (Figure 7). It was suspected that these may be “unnatural” oxidation products of the purportedly unstable 7,8-didehydroprotoberberines **1** and **2**, as per the observations of Paulsen and co-workers.^26^ To test this, the concentrations of **1**–**5** in *P. setiferum* capsules either stored at −20 °C or oven-dried at 40 °C for 2 weeks were compared (Figure 8). Interestingly, compounds **1** and **2** only showed a minor decrease in concentration in the oven-dried samples. Compound **5** remained below the level of detection, even in the oven-dried samples (S/N > 3). Compound **3** showed a significant increase in concentration in the oven-dried samples, approximately doubling, suggesting that **1** is more prone to oxidation than **2** (Figure 8). These surprising results indicate not only that the 7,8-didehydroprotoberberines (**1** and **2**) isolated herein are relatively stable but also that their oxidation to **3** and **5**—particularly the oxidation of **2** to **5**—may be enzymatically catalyzed by the plant in its later stages of life. Comparison of these results with those of Paulsen et al., who reported complete oxidation of 13,14-dihydrocoptisine after oven-drying of *C. majus* material,^26^ indicates that **1** and **2** are significantly more stable than 13,14-dihydrocoptisine.

### Cholinesterase Inhibitory Activities

All compounds were screened against a suite of cholinesterases of human and animal origin (Table 2). The protoberberine-type alkaloids (**1**–**4**) were active against both AChEs, with IC_50_ values between 3.4 and 10.3 μM against electric eel AChE (eeAChE), which was comparable to the activity of the positive control, neostigmine methylsulfate (Table 2). The activities against human recombinant AChE (hrAChE) were somewhat lower, with IC_50_ values ranging from 18.9 μM (**2**) to 66.6 μM (**1**). Compound **1** showed approximately 3-fold lower activity against AChEs when compared to **2** and 6-fold lower inhibition constants (Table 2), indicating that the presence of a 1,3-dioaxane group may be favorable for AChE inhibitory activity. The activities of **1**–**4** against hsBChE were lower on comparing to AChEs; the IC_50_ values ranged from 63.1 μM (**3**) to 104 μM (**4**) (Table 2). Dixon plot analysis (Supporting Information, Figures S32–S35) shows that **1**–**4** all behaved as competitive inhibitors of the three tested enzymes, indicating their interaction with the active site located in the enzyme gorge. Thus, it appears that the protoberberine scaffold leads to low μM eeAChE inhibitors regardless of the C-ring oxidation state. Due to the rarity and relatively recent discovery of the natural products featuring the 7,8-didehydroprotoberberine backbone, only a single report mentions their AChE inhibitory activities. Tsai and Lee (2010)^25^ reported two such compounds isolated from *Annona glabra* with modest activity against eeAChE. The most active of the two (IC_50_ = 30.8 μM) contained a 10-hydroxy-2,3,9-trimethoxy substitution pattern. Interestingly, the corresponding protoberberine alkaloid with an oxidized C ring exhibited a 3-fold drop in activity.^25^

**Table 1:** NMR Spectroscopic Data for 7,8-Didehydromecambridine TFA Salt (**1**) and 7,8-Didehydroorientalidine TFA Salt (**2**) in DMSO-*d*_6_

	7,8-didehydromecambridine TFA salt (**1**)			7,8-didehydroorientalidine TFA salt (**2**)	
position	δ_C_,^a^ type	δ_H_^b^ (*J* in Hz)	HMBC^c^	δ_C_,^a^ type	δ_H_^b^ (*J* in Hz)
1	139.6, C			139.5, C	
2	134.9, C			134.8, C	
3	148.7, C			148.7, C	
4	103.0, CH	6.69, s	1, 2, 3, 5, 14a	102.9, CH	6.68, s
4a	128.3, C			128.4, C	
5	28.7, CH_2_	α 3.00, ddd (16.0, 3.1, 2.5)	4, 4a, 6, 14a	28.7, CH_2_	α 2.98, ddd (16.0, 2.8, 2.1)
		β 3.18, ddd, (16.0, 13.1, 3.7)	1, 2, 4, 4a, 6, 14a		β 3.15, ddd (16.0, 12.5, 4.1)
6	54.8, CH_2_	α 3.96, m	4a, 5, 8, 14	54.6, CH_2_	α 3.94, m
		β 4.42, m	4a, 5, 8		β 4.39, m
7					
8	165.7, CH	9.29, dd (3.5, 1.8)	6, 8a, 9, 12a, 14	165.3, CH	9.23, dd (2.4, 1.6)
8a	120.5, C			117.1, C	
9	115.9, CH	7.60, s	8, 8a, 10, 11, 12a, 13	114.0, CH	7.50, s
10	151.7, C			147.3, C	
11	154.5, C			149.8, C	
12	133.2, C			119.9, C	
12a	133.7, C			129.7, C	
12α	53.8, CH_2_	4.60, d (11.7)	11, 12, 12a	63.4, CH_2_	5.04, d (15.0)
		4.54, d (11.7)	11, 12, 12a		4.89, d (15.0)
13	29.1, CH_2_	α 3.82, dd (17.0, 4.9)	8a, 12, 12a, 14	27.6, CH_2_	α 3.27, dd (17.0, 5.1)
		β 2.78 (dd, 17.0, 17.0)	8, 8a, 10, 11, 12, 12a, 14, 14a		β 2.68, dd (17.0, 16.8)
14	55.2, CH	5.27, m	4a, 6, 14a	54.6, CH	5.25, m
14a	117.1, C			116.9, C	
MeO-1	59.7, CH_3_	4.04, s	1	59.6, CH_3_	4.05, s
2-OCH_2_O-3	101.4, CH_2_	6.07, d (1.0)	2, 3	101.4, CH_2_	6.06, d (1.0)
		6.02, d (1.0)	2, 3		6.01, d (1.0)
MeO-10	56.3, CH_3_	3.90, s	10	56.2, CH_3_	3.87, s
MeO-11	61.6, CH_3_	3.90, s	11		
11-OCH_2_O-12α				91.3, CH_2_	5.48, d (5.8)
					5.40, d (5.8)

a 150 MHz.

b 600 MHz.

c HMBC correlations are from proton(s) stated to the indicated carbon.

**Table 2:** Cholinesterase Inhibitory Activities of Natural Products Isolated from the Capsules of *P. setiferum*^a^

compound	eeAChE IC_50_ (μM)	eeAChE *K*_i_ (μM)	hrAChE IC_50_ (μM)	hrAChE *K*_i_ (μM)	hsBChE IC_50_ (μM)	hsBChE *K*_i_ (μM)
**1**	10.3 ± 1.1	7.0	66.6 ± 2.4	31.0	100 ± 5	58.5
**2**	3.4 ± 4.7	1.0	18.9 ± 1.8	6.3	98.5 ± 0.6	47.0
**3**	6.8 ± 4.5	3.6	20.0 ± 0.9	13.2	63.1 ± 0.5	43.7
**4**	5.0 ± 1.0	2.0	19.9 ± 1.1	13.7	104 ± 4	35.5
**5**	n.a.	n.a.	n.a.	n.a.	n.a.	n.a.
**6**	260 ± 1	68.0	/	/	2.8 ± 3.0	0.6
**7**	/	/	/	/	7.1 ± 2.7	2.2
**8**	/	/	/	/	342 ± 3	/
**9**	/	/	/	/	221 ± 1	/
**10**	/	/	/	/	335 ± 4	/
**11**	/	/	/	/	/	/
neostigmine methylsulfate	6.0 ± 1.1	n.a.	n.a.	n.a.	92.7 ± 2.2	n.a.

a eeAChE = electric eel acetylcholinesterase, hrAChE = human recombinant acetlycholinesterase, hsBChE = horse serum butyrylcholinesterase /= inactive, n.a. = not assayed. IC_50_ = concentration required to induce 50% inhibition of enzyme activity; *K*_i_, inhibition constants, except for **6**, were determined for compounds with IC_50_ < 200 μM. Data are means ± SEM of three independent measurements. Compound **5** was not screened because of insufficient material.

The protoberberine scaffold is a known chemotype for AChE inhibition.^46^ For example, Ingkaninan and co-workers (2006)^47^ have reported the inhibitory activities against eeAChE for a range of protoberberine alkaloids isolated from *Stephania venosa* (Menispermaceae), as well as for known inhibitors berberine, palmatine, and jatrorrhizine.^47^ They found that compounds featuring a quaternary nitrogen center (i.e., pyridinium or *N*-methyl) were potent inhibitors (low- or sub-μM IC_50_ values), whereas the corresponding tertiary amines were relatively inactive (IC_50_ values >100 μM). Similar trends have been noted more recently by Chaichompoo et al.^48^ These contrast with the present results, where **4** provided a similar inhibitory profile to **1**–**3**; however the possibility that C-ring oxidation occurred between characterization and biological testing cannot be excluded. It must also be noted that the presently tested compounds contain different substitution patterns on the aromatic A and D rings in comparison to previously reported protoberberine AChE inhibitors, which may also influence their biological activity.^47,49^ While compounds **6** and **7** were inactive against AChEs, they both showed good activity against horse serum BChE (hsBChE) (IC_50_ values = 2.8 ± 3.0 and 7.1 ± 2.7 μM, respectively), indicating a good selectivity for BChE. Similar selectivity for human serum BChE compared to AChE has been previously reported for **6**.^50^ Compounds **8**–**11** all showed poor inhibition of the tested cholinesterases. It is of note that while (+)-salutaridine (**10**) is inactive,^51^ its enantiomer is active against both eeAChE and hsBChE.^48^ In summary, this investigation has identified several competitive inhibitors of cholinesterases with potencies in the low micromolar range (**1**–**4**, **6**, **7**). Further synthetic studies are underway to further investigate structure–activity relationships of these alkaloids.

## Experimental Section

### General Experimental Procedures

Optical rotations were recorded on a PerkinElmer 241 polarimeter (PerkinElmer, Waltham, MA, USA), and [α]_D_ values are given in 10^–1^ deg cm^2^ g^–1^. UV spectra were recorded on a NanoDrop One spectrophotometer (Thermo Fisher Scientific, Wilmington, DE, USA). ECD spectra were recorded using a J-1500 CD spectrometer (JASCO, Tokyo, Japan). NMR spectra were acquired at 298 K using a Bruker 600 MHz (TCI CRPHe TR-1H and 19F/13C/15N 5 mm-EZ CryoProbe) spectrometer (Bruker, Billerica, MA, USA). Chemical shifts were referenced to the solvent peak for (CD_3_)_2_SO at δ_H_ 2.50 and δ_C_ 39.52. High-resolution accurate mass measurements were performed by ESIMS using an LTQ-Velos Pro Orbitrap mass analyzer (Thermo Fisher Scientific, Waltham, MA, USA) equipped with an Agilent 1100 autosampler (Agilent, Santa Clara, CA, USA). High-resolution MS/MS and DESI data were collected on a Waters Xevo G2-XS quadrupole time-of-flight mass spectrometer (Waters Corp. Milford, MA, USA). Time series analyses were performed using ESI-MS in a linear ion trap (LTQ) mass spectrometer and a Surveyor Autosampler Plus autosampler (Thermo Fisher Scientific, Waltham, MA, USA). MPLC was performed on an ÄKTA FPLC using a P-920 pump, equipped with a UPC-900 UV detector and a Frac-900 fraction collector (Amersham Biosciences, Amersham, Buckinghamshire, UK). HPLC was performed with a Varian Pro Star pump (Varian, Crawley, UK). Acetonitrile (99.9%), dichloromethane (99.9%), and methanol (99.9%) were purchased from VWR (VWR International, Radnor, PA, USA). DMSO-*d*_6_ (99.8%) and methanol-*d*_4_ (99.5%) were from Apollo Scientific (Bredbury, UK). Water was Millipore Milli-Q PF filtered, and TFA was acquired from Iris Biotech GmBH (Marktredwitz, Germany). HPLC columns and C_18_ silica gel used to adsorb extracts before MPLC/HPLC (Sepra C18-E, 50 μm, 65 Å) were from Phenomenex (Torrance, CA, USA). Flash columns (Sfär C_18_, 12 g, 100 Å, 30 μm) were obtained from Biotage (Uppsala, Sweden). Filters used for LC-MS analysis were 0.2 μm PTFE membrane filters (VWR International, Radnor, PA, USA).

### Plant Material

*Papaver setiferum* material was collected twice from the same specimen from private land in Gottsunda, Uppsala, Sweden, on July 27, 2020 (aerial parts for preparative HPLC), on June 8, 2021 (aerial parts for voucher specimen), and between June 8 and July 7, 2021 (aerial parts for time-series and DESI-IMS). The plant material was identified by Mats L. Hjertson (Uppsala University). The voucher specimen (Global Universal Identifier: UPS:BOT-V-998275) is housed within the Museum of Evolution at Uppsala University, and an image of the voucher is provided in the Supporting Information (Figure S36, Supporting Information).

### Extraction and Isolation

The capsules of *P. setiferum* were oven-dried (40 °C, 22 h), ground to a powder (1.6 g), and extracted exhaustively via sonication in MeOH/CH_2_Cl_2_ (400 mL) and MeOH (800 mL). The solution was filtered and evaporated to dryness, yielding 0.9 g of extract. This extract (0.9 g) was adsorbed onto C_18_ silica gel (0.9 g), and the extract-impregnated gel was loaded onto a C_18_ flash column (Biotage Sfär C_18_, 12 g, 100 Å, 30 μm). The column was then eluted at a flow rate of 10 mL/min with 100% H_2_O (0.1% TFA) for 5 min, then to 100% CH_3_CN (0.1% TFA) over 50 min. The column was then eluted with 100% CH_3_CN (0.1% TFA) for an additional 5 min. Sixty fractions were collected at 1 min intervals and labeled A.1–A.60. Fraction A.17 (40 mg) and A.23–A.26 (25 mg) were put aside for further purification. Fractions A.18–A.20 contained isothebaine (**6**) (30 mg).

Fractions A.23–A.26 (25 mg) were recombined and further purified by C_18_ HPLC (Kinetex XB-C_18_, 5 μm, 100 Å, 21.2 × 150 mm) at a flow rate of 9 mL/min using a gradient from 95% H_2_O (0.1% TFA)/5% CH_3_CN (0.1% TFA) to 85% H_2_O (0.1% TFA)/15% CH_3_CN (0.1% TFA) over 5 min, then to 65% H_2_O (0.1% TFA)/35% CH_3_CN (0.1% TFA) over 90 min. Ninety-five fractions were collected and labeled B.1–B.95. Fractions B.41–B.44 contained **1** (3 mg), and fractions B.57–B.63 contained **2** (4 mg). Due to their suspected instability, **1** and **2** were stored at −20 °C in glass vials flushed with nitrogen. Fractions B.46–B.52 (8 mg) were further purified using biphenyl HPLC (Kinetex Biphenyl, 5 μm, 100 Å, 10.0 × 250 mm) using a gradient from 95% H_2_O (5 mM NH_4_OAc, pH 4.8)/5% CH_3_CN to 62% H_2_O (5 mM NH_4_OAc, pH 4.8)/38% CH_3_CN over five min, then to 58% H_2_O (5 mM NH_4_OAc, pH 4.8)/42% CH_3_CN over 20 min, then to 5% H_2_O (5 mM NH_4_OAc, pH 4.8)/95% CH_3_CN over 5 min. The flow used was 4 mL/min. Thirty fractions were collected at 1 min intervals and labeled C.1–C.30. Fractions C.4–C.5 contained **3** (1.7 mg), and fractions C.13–C.14 contained **4** (2.6 mg). Fraction C.12 contained a mixture of **4** and **5** (1:1) and upon being left in an NMR tube for four months, contained pure **5**, presumably by the complete oxidation of **4** to **5**.

Fraction A.17 (40 mg) was further separated by C_18_ HPLC (Kinetex XB-C_18_, 5 μm, 100 Å, 21.2 × 250 mm) using a gradient from 95% H_2_O (0.1% TFA)/5% CH_3_CN (0.1% TFA) to 80% H_2_O (0.1% TFA)/20% CH_3_CN (0.1% TFA) over 76 min, then to 5% H_2_O (0.1% TFA)/95% CH_3_CN (0.1% TFA) over 10 min. Eighty-six fractions were collected and labeled D.1–D.86. Fraction D.44 contained **11** (3.1 mg), fraction D.48 contained **8** (1.1 mg), fractions D.60 and D.61 contained **10** (2.1 mg), fractions D.64–D.67 contained **9** (1.7 mg), and fractions D.70 and D.71 contained **7** (1.2 mg). For a detailed flowchart of isolation procedures, see the Supporting Information (S37).

#### 7,8-Didehydromecambridine (**1**) TFA salt:

yellow, amorphous solid; [α]_D_^25^ −102 (*c* 0.14, MeOH); UV (MeOH) λ_max_ (log ε) 367 (3.48), 308 (3.80), 245 (4.02), 211 (4.42) nm; ECD (*c* 0.025 mM, MeOH) λ_max_ (Δε) 355 (−1.0), 324 (0.0), 313 (+0.3), 299 (0.0), 279 (−0.7) 216 (−9.66), 204 (0.0), 200 (+2.6) nm; ^1^H and ^13^C NMR data, Table 1; HRESIMS *m*/*z* [M]^+^ 398.1597 (calcd for C_22_H_24_NO_6_^+^, 398.1598).

#### 7,8-Didehydroorientalidine (**2**) TFA salt:

yellow, amorphous solid; [α]_D_^25^ −43 (*c* 0.12, MeOH); UV (MeOH) λ_max_ (log ε) 300 (3.08), 251 (3.17), 211 (3.78) nm; ECD (*c* 0.05 mM, MeOH) λ_max_ (Δε) 392 (0.0), 300 (−1.3) 213 (−5.8) nm; ^1^H and ^13^C NMR data, Table 1; HRESIMS *m*/*z* [M]^+^ 396.1441 (calcd for C_22_H_22_NO_6_^+^, 396.1442).

### Computational Methods

An extensive conformer search was performed on **1** and **2** using the Monte Carlo Minimum method (MCMM) and the molecular mechanics OPLS3 force field using Schrödinger MacroModel 2016 software (Schrödinger, LLC, New York, NY, USA). The subsequent conformational suites were then subjected to initial density functional theory (DFT) geometry optimization at the B3LYP-6-31G(d) level of theory, followed by a second optimization at the higher B3LYP-6-311++(d,p) level of theory using Gaussian 16.^52^ The resultant geometry-optimized conformers were then filtered for duplicate, high-energy (>3.0 kcal/mol) and saddle point conformers before electronic transition and rotational strength calculations were performed using TDDFT at the B3LYP/6-311++G(d,p) level. The polarizable continuum model (PCM) was included in calculations for both the second geometry optimization DFT and TDDFT ECD calculations.^53^ The weighted average of the calculated UV and ECD spectra was performed using a Boltzmann distribution function within the freely available SpecDis software (version 1.71).^54^ The TDDFT-calculated UV and ECD spectra for **1** and **2** were matched with their respective experimental UV and ECD data using Gaussian band shapes (sigma/gamma values of 0.28 and 0.26 eV) and UV corrections of +15 and −6 nm, respectively. Automation processes with the Gowonda HPC were carried out using Windows 10 OS and modified python scripts based upon the recently addended Willoughby protocol.^55,56^

### Desorption Electrospray Ionization Imaging Mass Spectrometry

*P. setiferum* capsules were cut in half along both the long and short axes shortly after collection, then snap frozen in liquid nitrogen. The pods were stored at −80 °C until being sectioned into 20 μm-thick sections using a cryostat microtome (Leica CM1900 UV, Leica Microsystems, Welzlar, Germany) and then thaw-mounted onto glass slides and air desiccated. The petals and leaves were dry pressed between sheets of paper. Subsequently, the fragments were cut with a scalpel and mounted onto glass slides using double-sided tape. DESI imaging was performed on a Waters Xevo G2-XS Quadrupole time-of-flight mass spectrometer equipped with a Waters DESI XS interface (Waters Corp.). The solvent (98% MeOH, 0.1% FA) was delivered at a rate of 3 μL/min using a Waters nanoAquity auxiliary solvent manager unit (Waters Corp.). The MS acquisition was performed in positive mode with a capillary voltage of 3 kV and with an acquisition window of 100–1000 *m*/*z*. The DESI interface was set up according to the manufacturer’s instructions with a needle to specimen angle of 75°. The reference points were defined using the Prosalia Omni Spray 2D v.2.1.0.2 software. The experiments were set up using Waters HDI v1.5 software and were implemented under the control of Waters MassLynx v4.2 (Waters Corp.). All specimens were analyzed with a lateral resolution of 50–200 μm and velocity of 50–100 μm/sec. Finally, the data were processed and analyzed using Waters HDI v1.5.

### Alkaloid LC-MS Analysis

The alkaloid analysis was based on De Vos et al. (2007).^57^*P. setiferum* material was collected at four time points: (1) approximately 3 days before flowering, (2) during flowering, (3) approximately 2 weeks after flowering, and (4) approximately 4 weeks after flowering (by which point the flowers had dried and turned brown). At each collection time, a sample of the aerial parts (three separate stems, each with several leaves and a capsule) was collected and stored at −20 °C. The samples were later separated into capsules, leaves, and stems and ground into a powder using a mortar and pestle. One hundred milligrams (±5 mg) of each was transferred into an Eppendorf tube, and each weight was recorded. Cold MeOH (0.1% FA) was added to each in a 1:10 w/v ratio, and the tubes were sonicated for 30 min. The samples were centrifuged for 5 min at 13 000 rpm, and the supernatant was filtered (0.2 μm). A pooled sample was prepared by taking 20 μL from each sample and combining into a single vial. All samples were diluted 1:10 in Milli-Q water prior to LC-MS analysis. The samples were randomly ordered for LC-MS, with the pooled sample run interspaced after every fourth sample. An injection volume of 2 μL was used for each sample. The solvents used were 5% CH_3_CN (0.1% FA) (solvent A) and 95% CH_3_CN (0.1% FA) (solvent B). The column used was a Phenomenex Kinetex XB-C_18_ (2.6 μm, 100 Å, 3.0 × 100 mm) with a linear solvent gradient from 15% to 50% solvent B over 20 min. Alkaloids were identified by comparison of retention times with authentic standards.

### Protoberberine Oxidation Analysis

From three *P. setiferum* capsules [taken from time point (1) in the time series analysis], two lots of 100 ± 5 mg of material were weighed into Eppendorf tubes. One set of samples was stored with the lids open in the oven at 40 °C, while the others were stored in the freezer at −20 °C. After 2 weeks, the samples were analyzed by LC-MS, as described above.

### Cholinesterase Inhibition Assay

Cholinesterase activities were measured by the Ellman method^58^ adapted for 96-well microtiter plates, as previously described.^59^ The compounds were first screened for the IC_50_ determination, and for those with IC_50_ < 200 μM, the inhibitory constants (*K*_i_) were determined. Stock solutions of the tested compounds (5 mg/mL) and of the positive control (1 mg/mL neostigmine bromide, Sigma-Aldrich, St. Louis, MO, USA) were prepared in 100% MeOH. These solutions were added to the wells and gradually diluted in 100 mM potassium phosphate buffer (pH 7.4) to the final volume of 50 μL. Acetylthiocholine chloride and 5,5′-dithiobis-2-nitrobenzoic acid were then dissolved in the same buffer to the respective final concentrations of 1 and 0.5 mM and added (100 μL) to the wells of the microtiter plates. eeAChE, hrAChE, or hsBChE (all Sigma-Aldrich) were dissolved in the same buffer to 0.0075 U/mL. A 50 μL amount of each of the cholinesterases was added to start the reactions, which were followed spectrophotometrically at 405 nm and 25 °C for 5 min using a kinetic microplate reader (Dynex Technologies Inc., Chantilly, VA, USA). Blank reactions without the inhibitors were run in the presence of the appropriate dilution of MeOH in 100 mM potassium phosphate buffer (pH 7.4). The concentrations of the compounds inducing 50% inhibition of enzyme activity (IC_50_) were determined and expressed as mean values ± SEM. To determine the inhibition constants (*K*_i_), the kinetics were monitored using three different final substrate concentrations (0.125, 0.25, 0.5 mM). Each measurement was repeated at least three times. Data were analyzed using OriginPro software (OriginPro 2020, OriginLab Corporation, Northampton, MA, USA).

## Supporting Information Available

Spectroscopic data for compounds **1**, **2**, and **5**, detailed isolation procedures, extra DESI-IMS data, Dixon plots for **1**–**4**, and an image of the voucher specimen (PDF)
