# Supplementary material for: Spatial Distribution and Stability of Cholinesterase Inhibitory Protoberberine Alkaloids from Papaver setiferum
Source: J Nat Prod. 2021 Dec 15;85(1):215–24. doi: 10.1021/acs.jnatprod.1c00980 (PMC8805119; doi:10.1021/acs.jnatprod.1c00980)
Supplement: Supplementary file 1 — np1c00980_si_001.pdf [file np1c00980_si_001.pdf]

Spatial Distribution and Stability of Cholinesterase Inhibitory Protoberberine Alkaloids from *Papaver setiferum* (Papaveraceae)

Neda Safa, Tomaž Trobec, Darren C. Holland, Blazej Slazak, Erik Jacobsson, Jeffrey A. Hawkes, Robert Frangež, Kristina Sepčić, Ulf Göransson, Lindon W. K. Moodie,* and Luke P. Robertson*

Supporting Information

Table of Contents

Figure S1. ^{1}H NMR spectrum (600 MHz) of **1** in DMSO-d_6
Figure S2. ^{1}H NMR spectrum (zoomed from 10.0-6.0 ppm) (600 MHz) of **1** in DMSO-d_6
Figure S3. ^{1}H NMR spectrum (zoomed from 5.5-2.0 ppm) (600 MHz) of **1** in DMSO-d_6
Figure S4. ^{13}C NMR spectrum (200 MHz) of **1** in DMSO-d_6
Figure S5. COSY NMR spectrum (600 MHz) of **1** in DMSO-d_6
Figure S6. ^{1}H and ^{13}C decoupled HSQC NMR spectrum (600 MHz) of **1** in DMSO-d_6
Figure S7. ^{1}H decoupled HSQC NMR spectrum (600 MHz) of **1** in DMSO-d_6
Figure S8. HMBC NMR spectrum (600 MHz) of **1** in DMSO-d_6
Figure S9. ROESY NMR spectrum (600 MHz) of **1** in DMSO-d_6
Figure S10. ^{1}H NMR spectrum (600 MHz) of **2** in DMSO-d_6
Figure S11. ^{1}H NMR spectrum (zoomed from 9.5-6.0 ppm) (600 MHz) of **2** in DMSO-d_6
Figure S12. ^{1}H NMR spectrum (zoomed from 5.5-2.0 ppm) (600 MHz) of **2** in DMSO-d_6
Figure S13. ^{13}C NMR spectrum (200 MHz) of **2** in DMSO-d_6
Figure S14. COSY NMR spectrum (600 MHz) of **2** in DMSO-d_6
Figure S15. ^{1}H and ^{13}C decoupled HSQC NMR spectrum (600 MHz) of **2** in DMSO-d_6
Figure S16. ^{1}H decoupled HSQC NMR spectrum (600 MHz) of **2** in DMSO-d_6
Figure S17. HMBC NMR spectrum (600 MHz) of **2** in DMSO-d_6
Figure S18. ROESY NMR spectrum (600 MHz) of **2** in DMSO-d_6
Figure S19. UV spectrum of **1** in MeOH (concentration: 0.01 mg/mL)
Figure S20. UV spectrum of **2** in MeOH (concentration: 0.02 mg/mL)
Figure S21. ECD spectrum of **2** in MeOH (concentration: 0.02 mg/mL)
Figure S22. MS/MS spectra of **1** and **2**
Table S23. NMR spectroscopic data for 7,8,13,14-dehydroorientalidine (**5**) in methanol-d_4
Figure S24. ^{1}H NMR spectrum (600 MHz) of **5** in methanol-d_4
Figure S25. ^{13}C NMR spectrum (150 MHz) of **5** in methanol-d_4
Figure S26. COSY NMR spectrum (600 MHz) of **5** in methanol-d_4
Figure S27. HSQC NMR spectrum (600 MHz) of **5** in methanol-d_4
Figure S28. HMBC NMR spectrum (600 MHz) of **5** in methanol-d_4
Figure S29. ROESY NMR spectrum (600 MHz) of **5** in methanol-d_4
Figure S30. Extra DESI-IMS data of *P. setiferum* capsules.
Figure S31. Isolation timeline of compounds **1**-**2**
Figure S32. Dixon plots for determination of type of inhibition and inhibition constants (K_i) for compound **1** against electric eel acetylcholinesterase (**A**), human recombinant acetylcholinesterase (**B**) and horse serum butyrylcholinesterase (**C**). Substrate concentrations: 0.125 mM (▲), 0.25 mM (●), 0.5 mM (■).

Figure S33. Dixon plots for determination of type of inhibition and inhibition constants (K_i) for compound **2** against electric eel acetylcholinesterase (**A**), human recombinant acetylcholinesterase (**B**) and horse serum butyrylcholinesterase (**C**). Substrate concentrations: 0.125 mM (▲), 0.25 mM (●), 0.5 mM (■).
Figure S34. Dixon plots for determination of type of inhibition and inhibition constants (K_i) for compound **3** against electric eel acetylcholinesterase (**A**), human recombinant acetylcholinesterase (**B**) and horse serum butyrylcholinesterase (**C**). Substrate concentrations: 0.125 mM (▲), 0.25 mM (●), 0.5 mM (■).
Figure S35. Dixon plots for determination of type of inhibition and inhibition constants (K_i) for compound **4** against electric eel acetylcholinesterase (**A**), human recombinant acetylcholinesterase (**B**) and horse serum butyrylcholinesterase (**C**). Substrate concentrations: 0.125 mM (▲), 0.25 mM (●), 0.5 mM (■).
Figure S36. Voucher specimen of *Papaver setiferum*.
Figure S37. Isolation flow chart of compounds **1**-**11.**

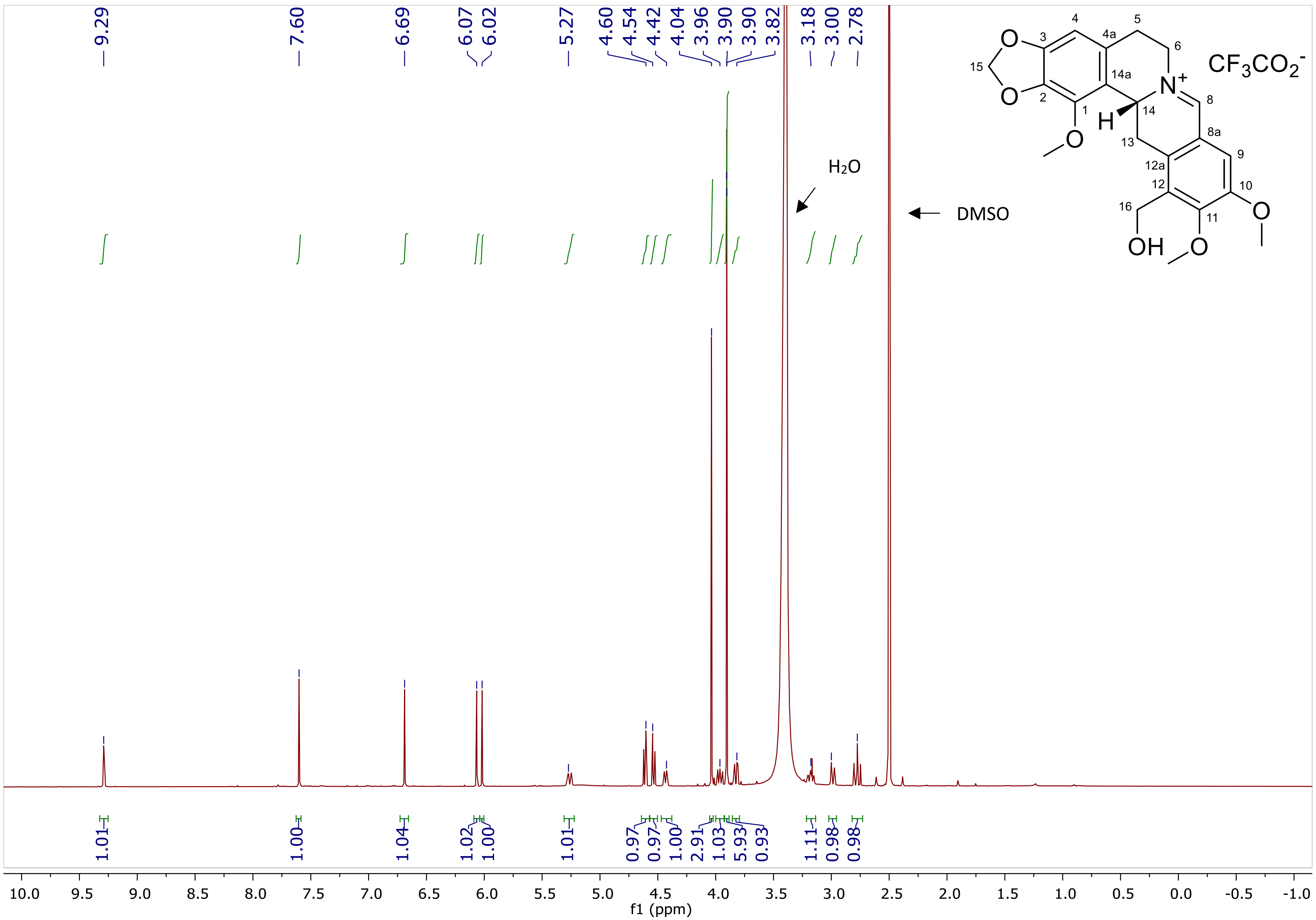

Figure S1. ^{1}H NMR spectrum (600 MHz) of **1** in DMSO-d_6

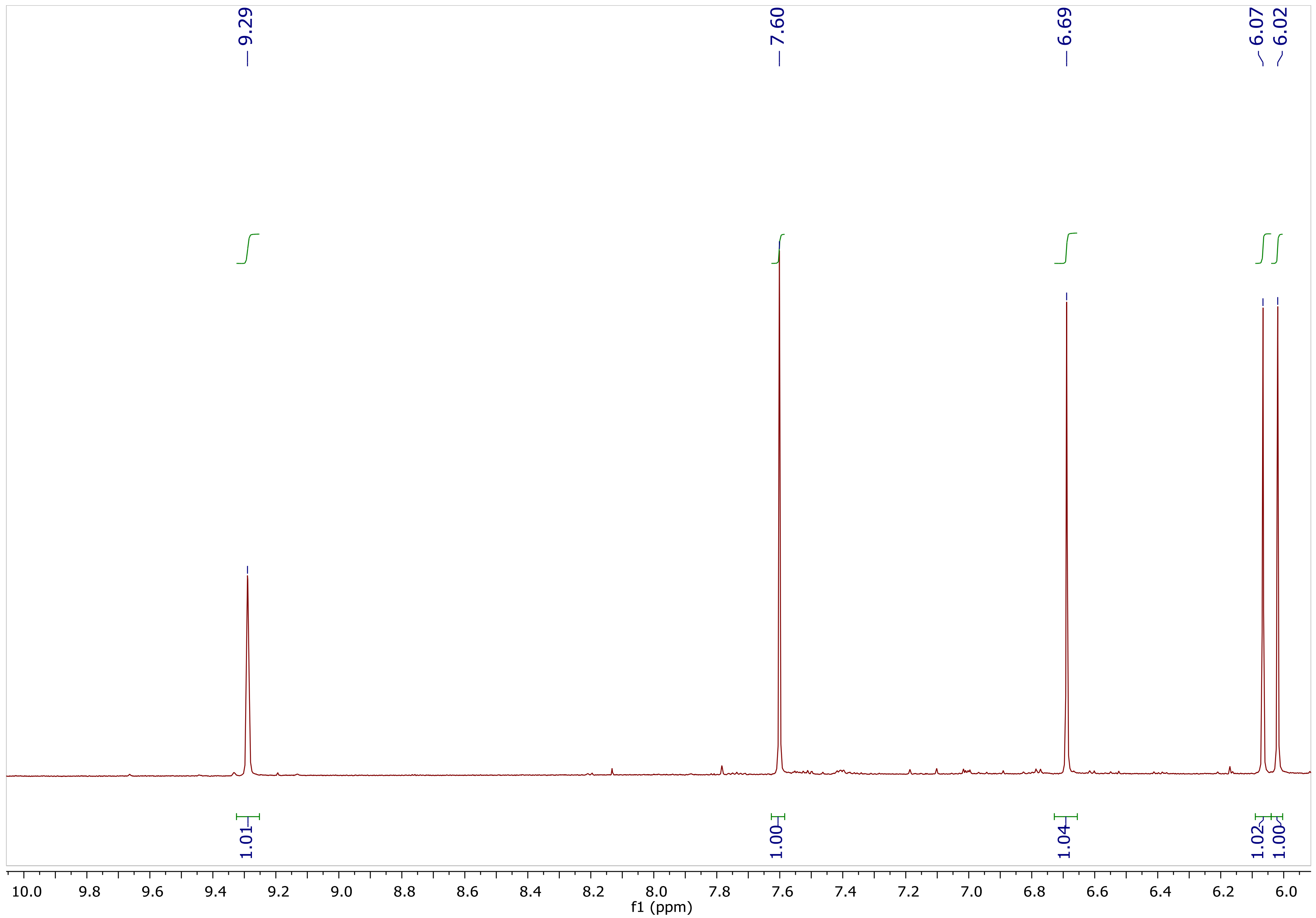

Figure S2. ^{1}H NMR spectrum (zoomed from 10.0-6.0 ppm) (600 MHz) of **1** in DMSO-d_6

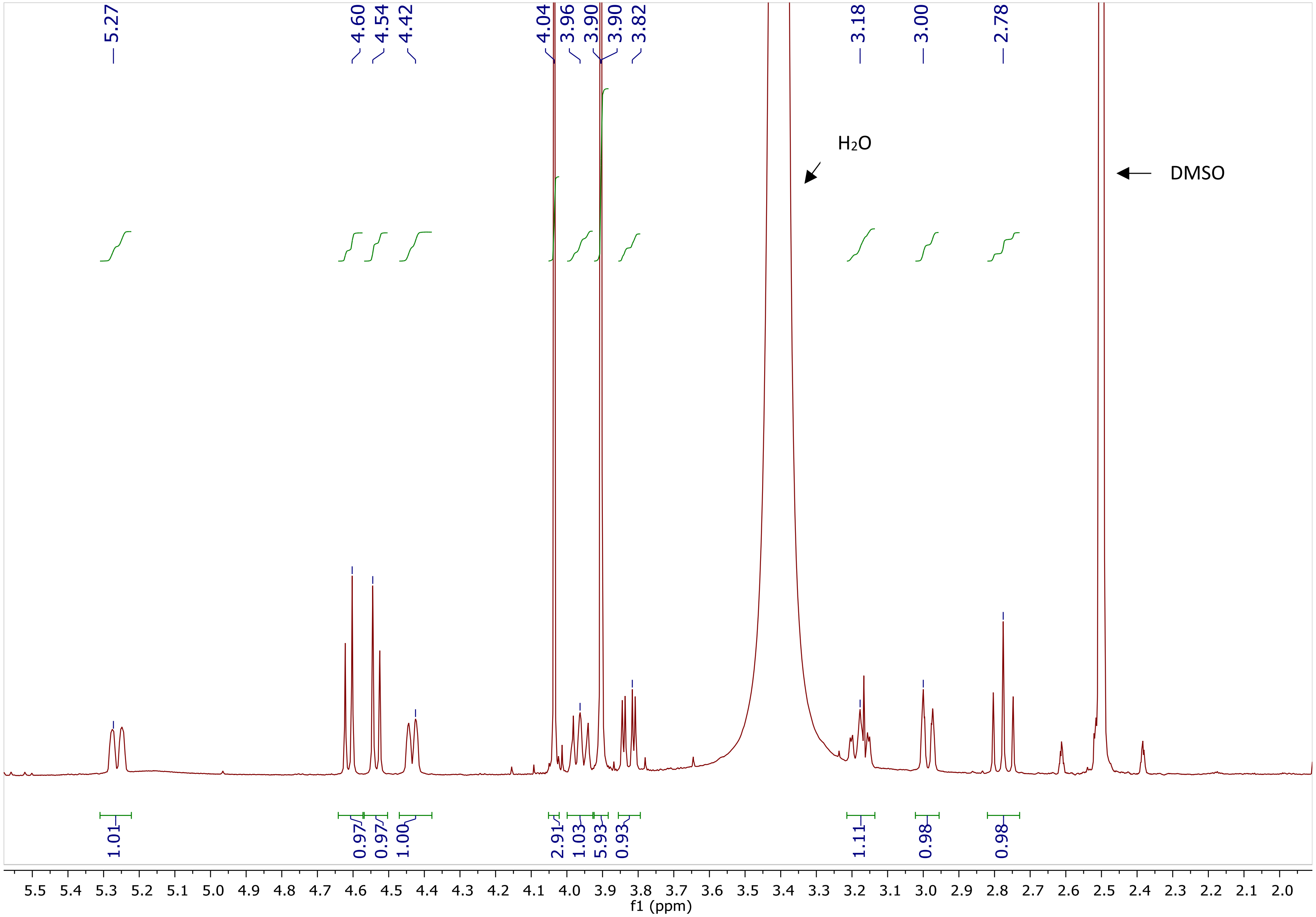

Figure S3. ^{1}H NMR spectrum (zoomed from 5.5-2.0 ppm) (600 MHz) of **1** in DMSO-d_6

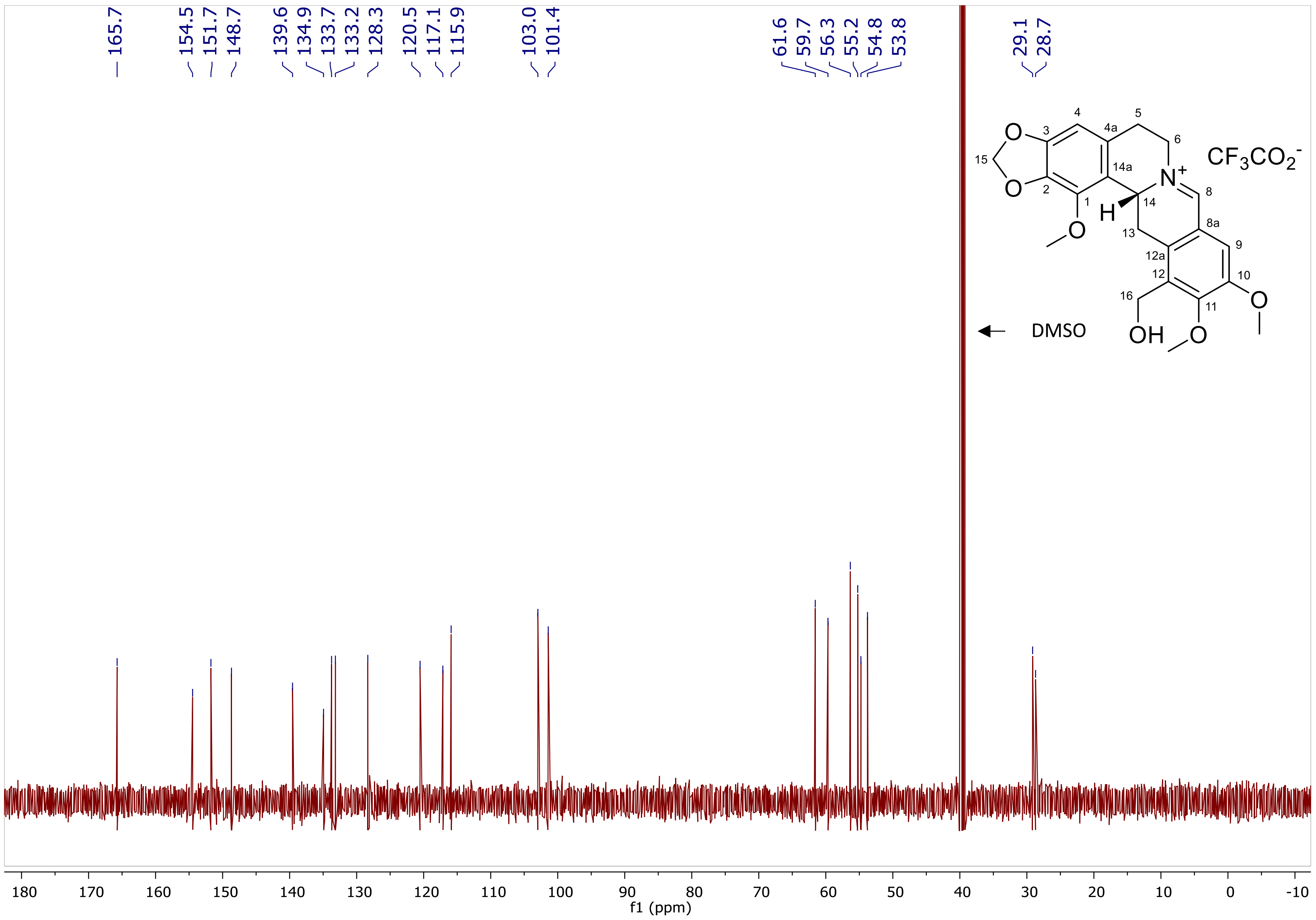

Figure S4. ^{13}C NMR spectrum (200 MHz) of **1** in DMSO-d_6

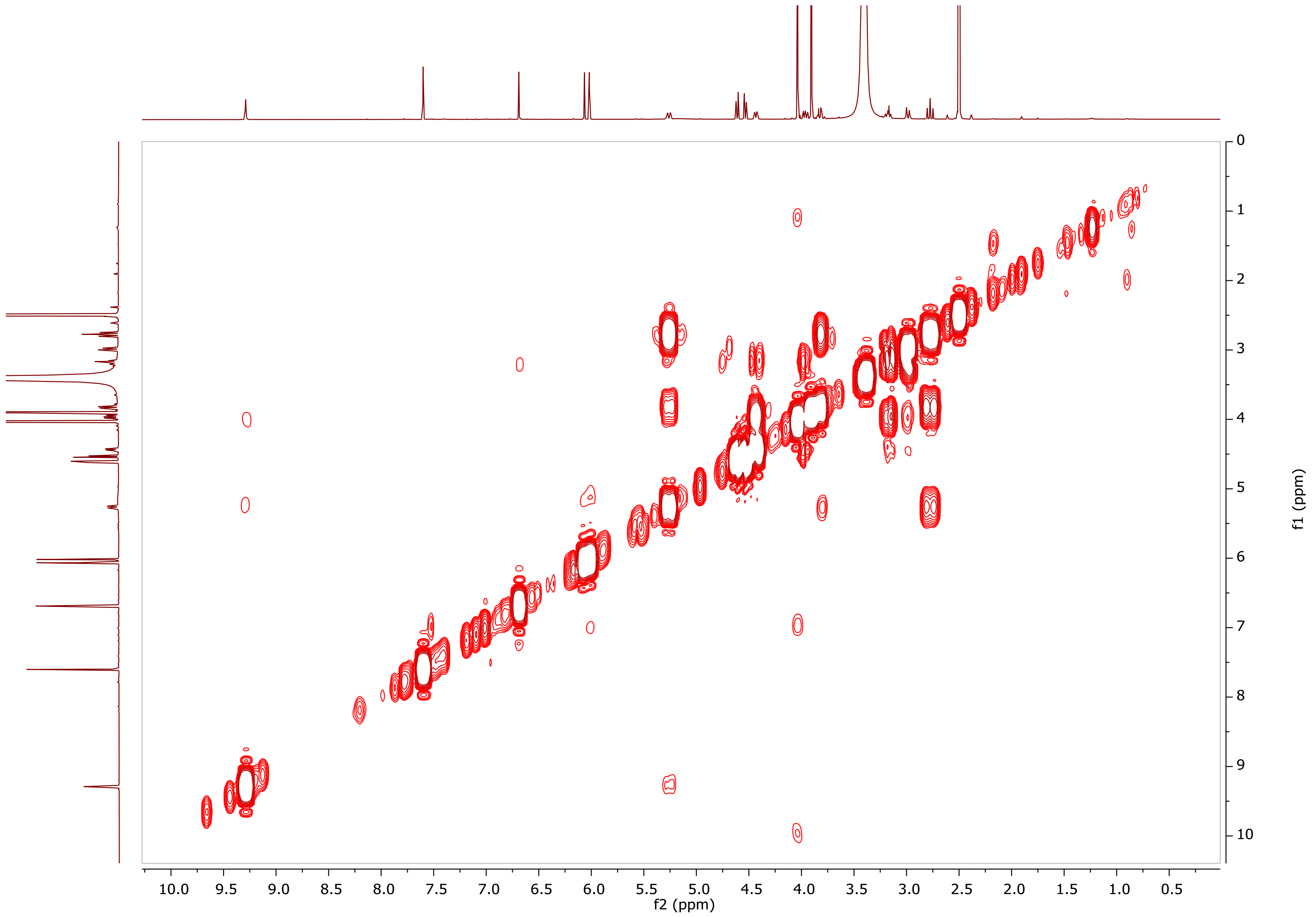

Figure S5. COSY NMR spectrum (600 MHz) of **1** in DMSO-d_6

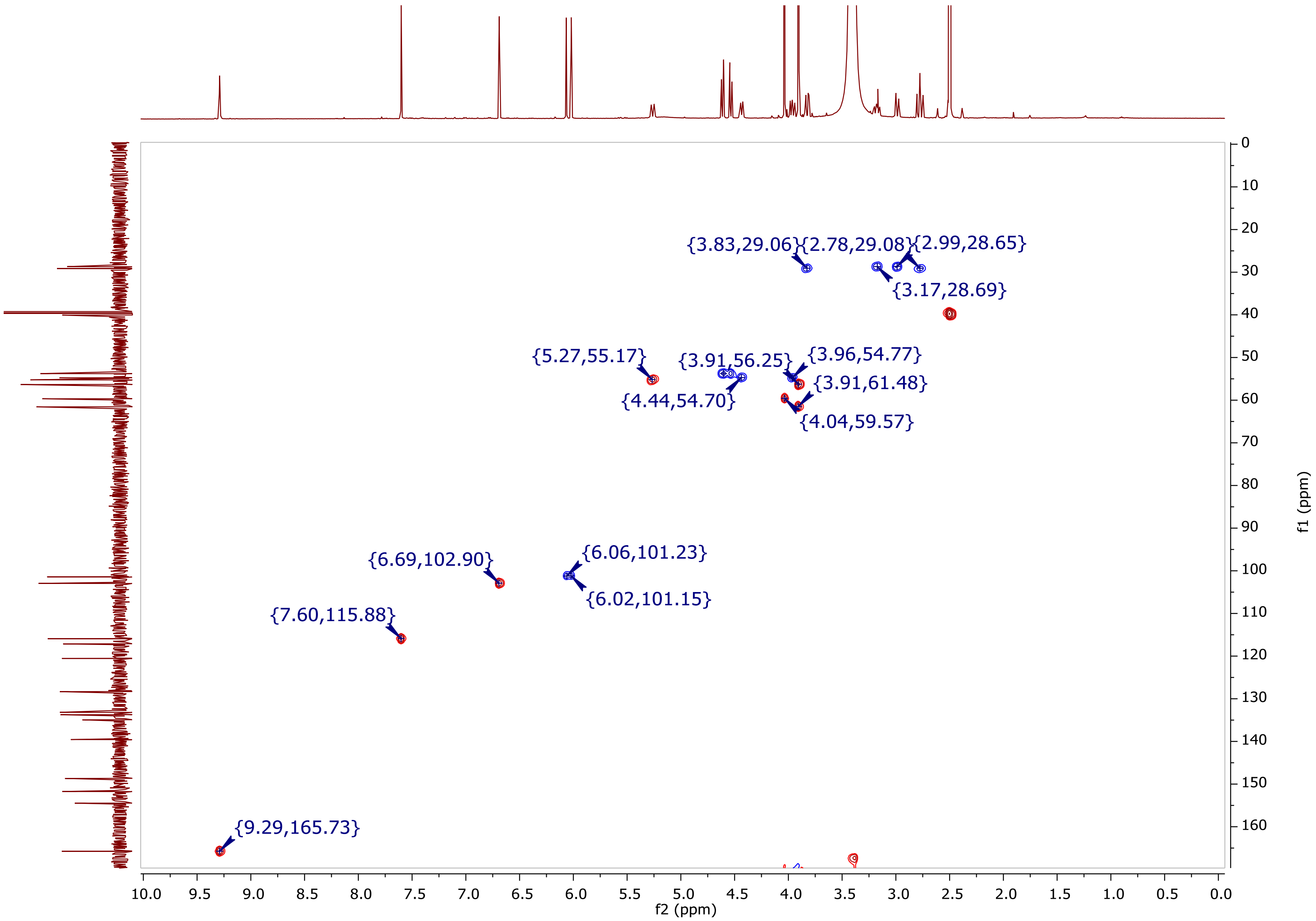

Figure S6. ^{1}H and ^{13}C decoupled HSQC NMR spectrum (600 MHz) of **1** in DMSO-d_6

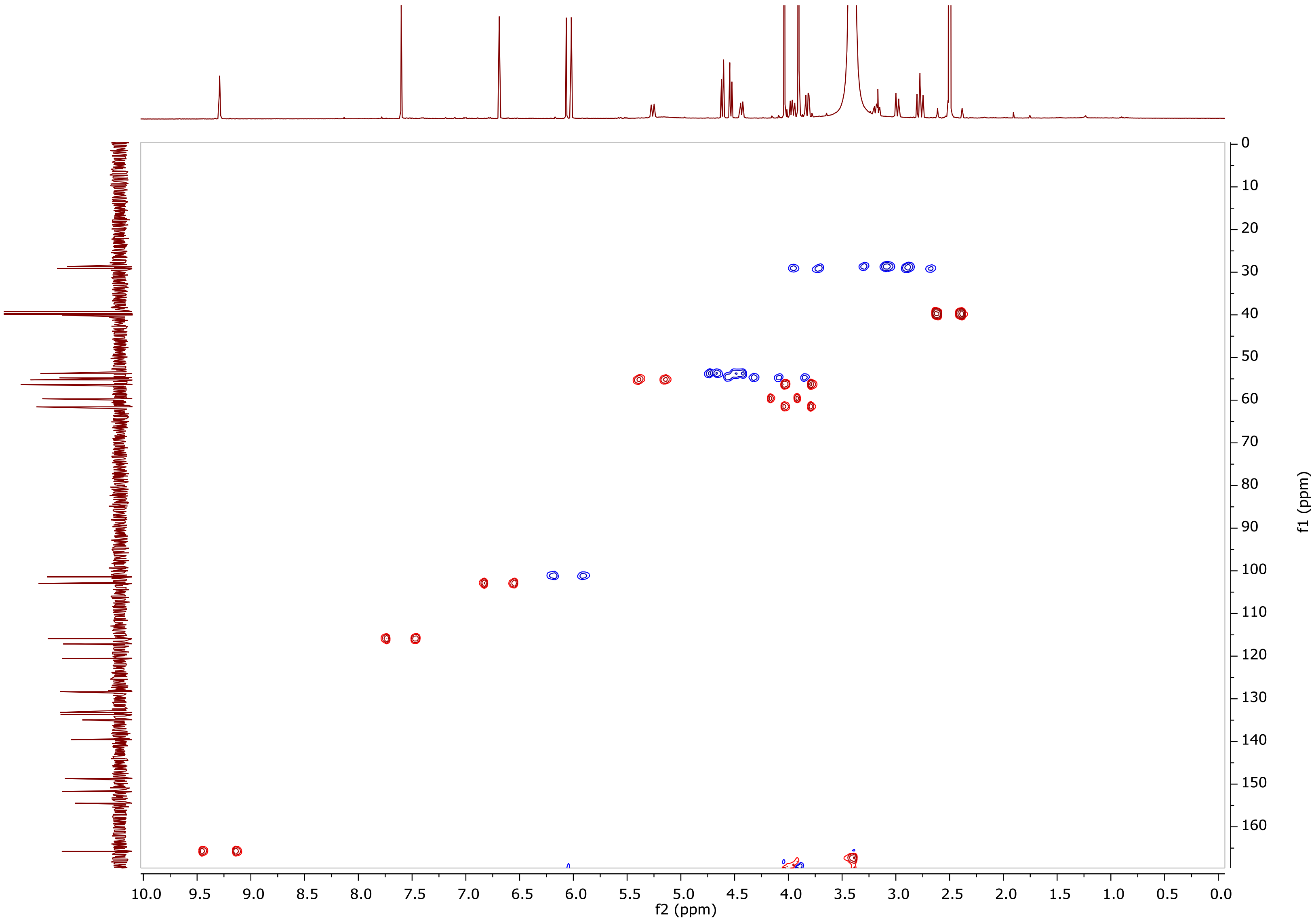

Figure S7. ^{1}H decoupled HSQC NMR spectrum (600 MHz) of **1** in DMSO-d_6

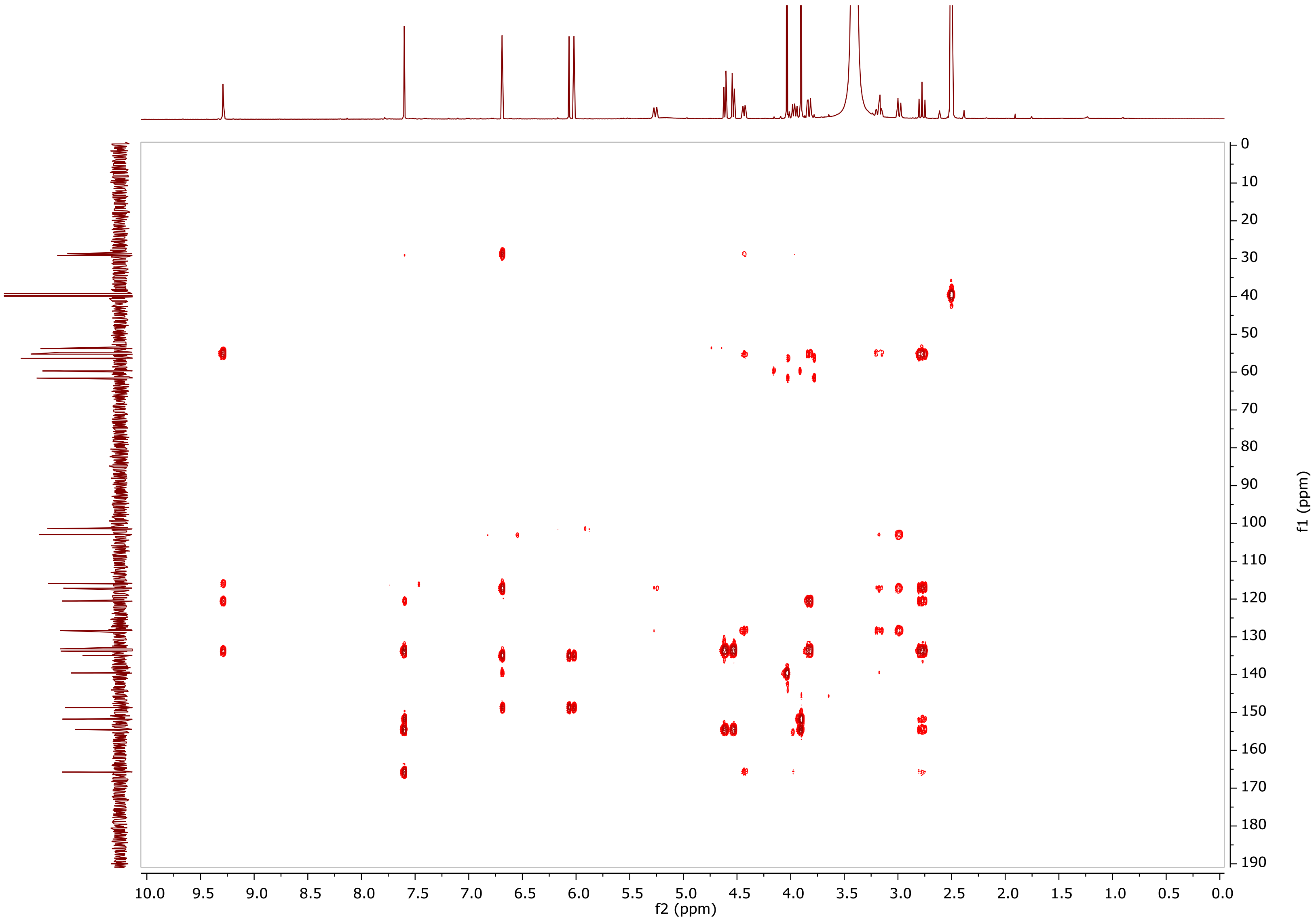

Figure S8. HMBC NMR spectrum (600 MHz) of **1** in DMSO-d_6

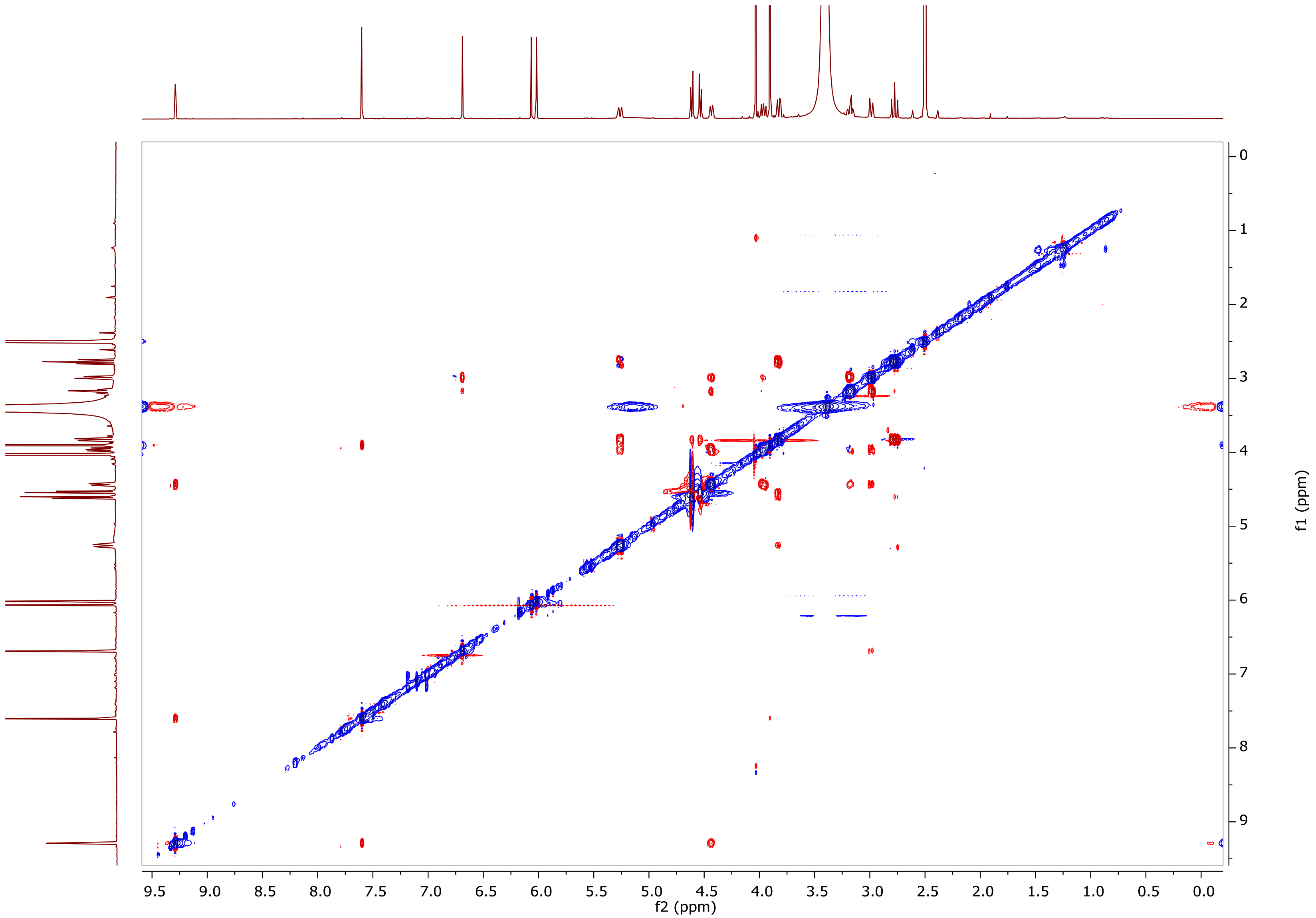

Figure S9. ROESY NMR spectrum (600 MHz) of **1** in DMSO-d_6

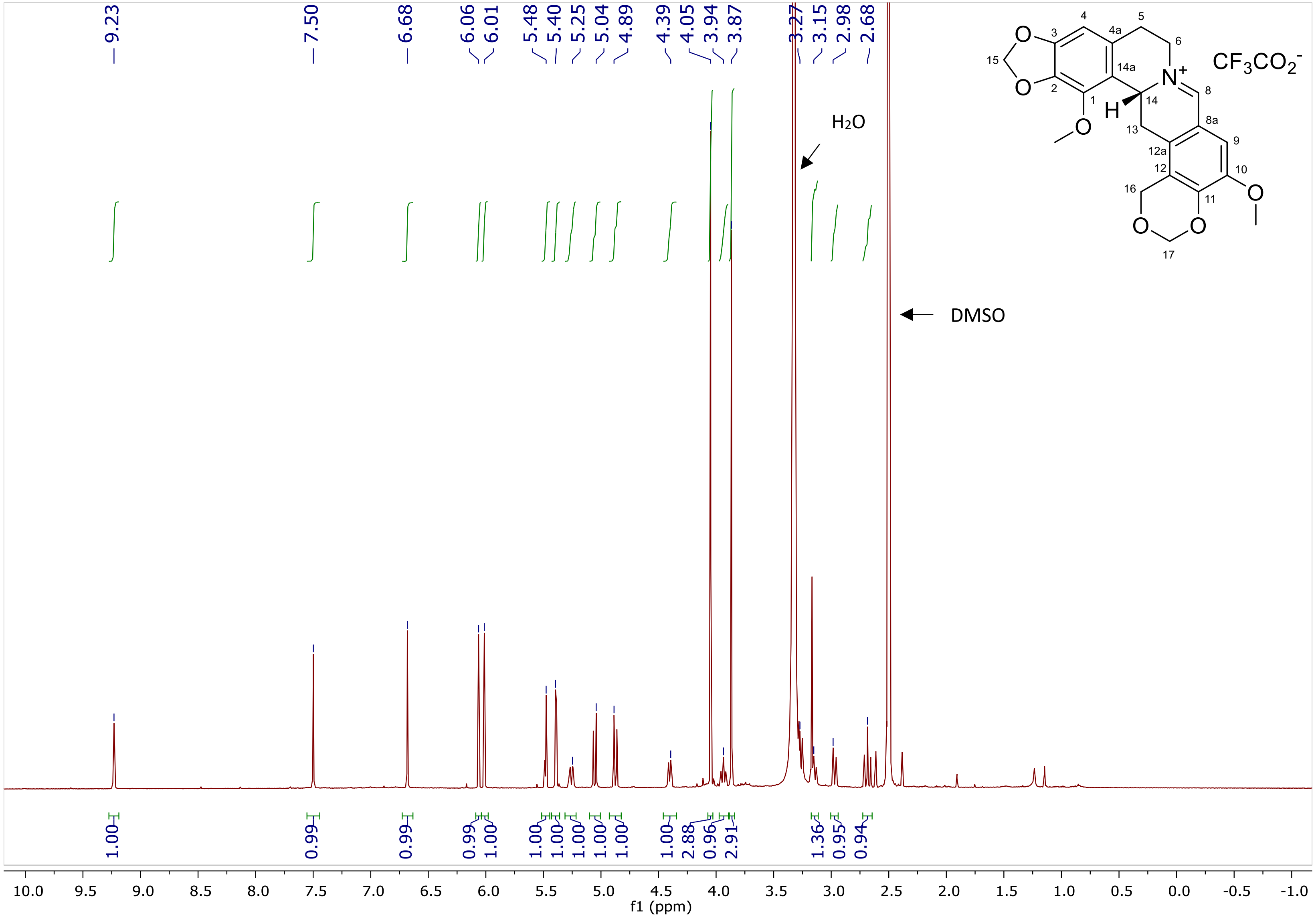

Figure S10. ^{1}H NMR spectrum (600 MHz) of **2** in DMSO-d_6

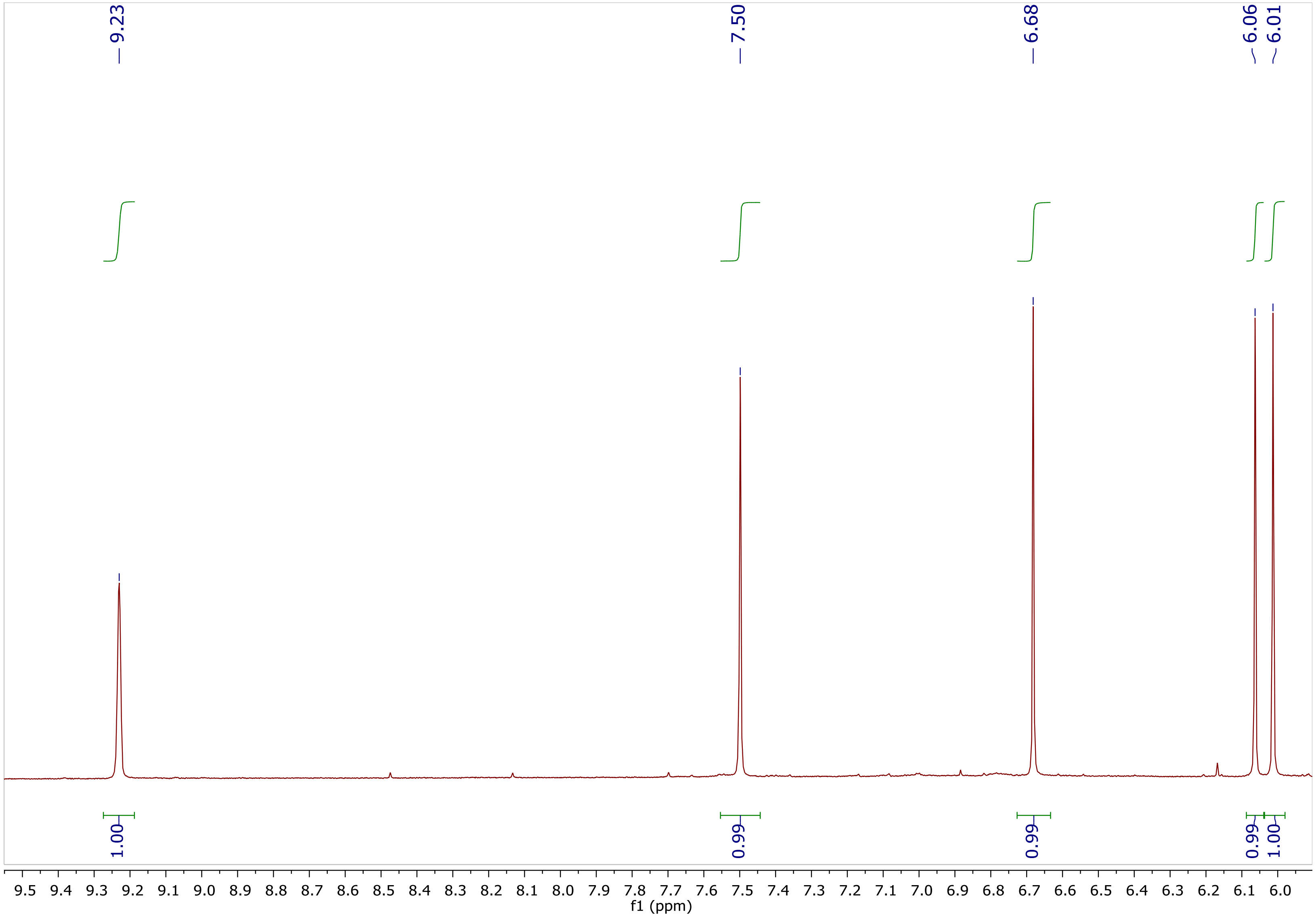

Figure S11. ^{1}H NMR spectrum (zoomed from 9.5-6.0 ppm) (600 MHz) of **2** in DMSO-d_6

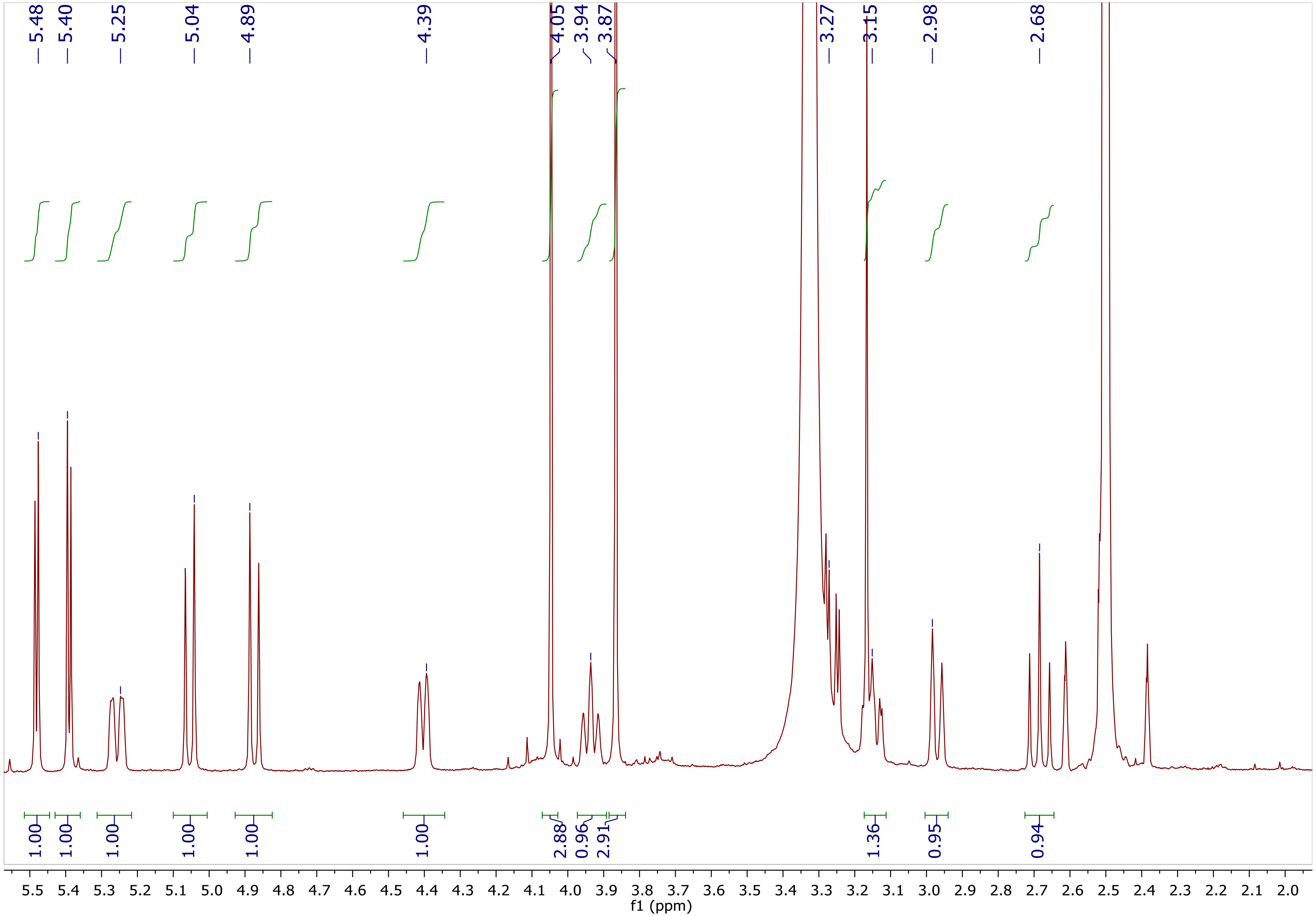

Figure S12. ^{1}H NMR spectrum (zoomed from 5.5-2.0 ppm) (600 MHz) of **2** in DMSO-d_6

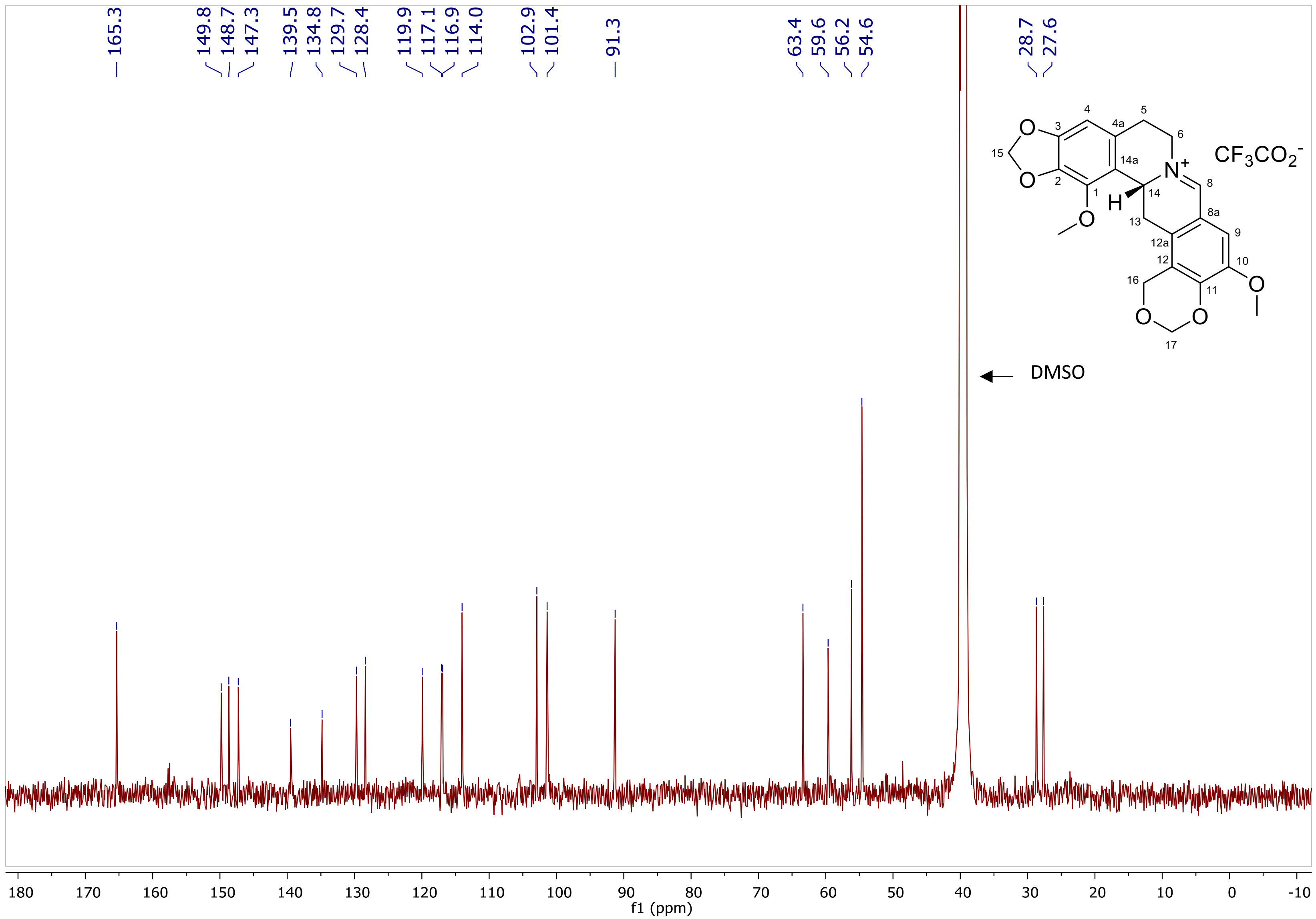

Figure S13. ^{13}C NMR spectrum (200 MHz) of **2** in DMSO-d_6

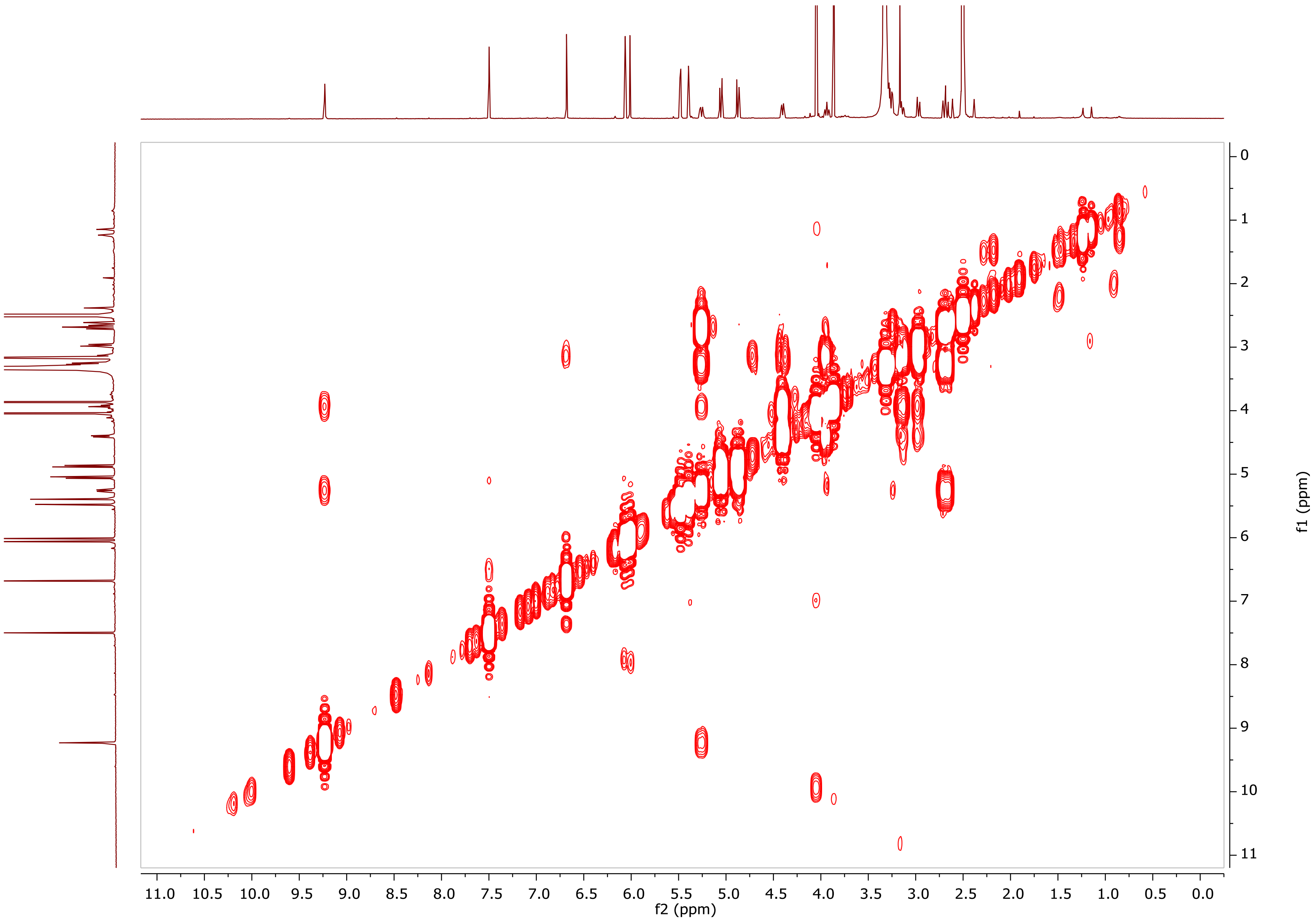

Figure S14. COSY NMR spectrum (600 MHz) of **2** in DMSO-d_6

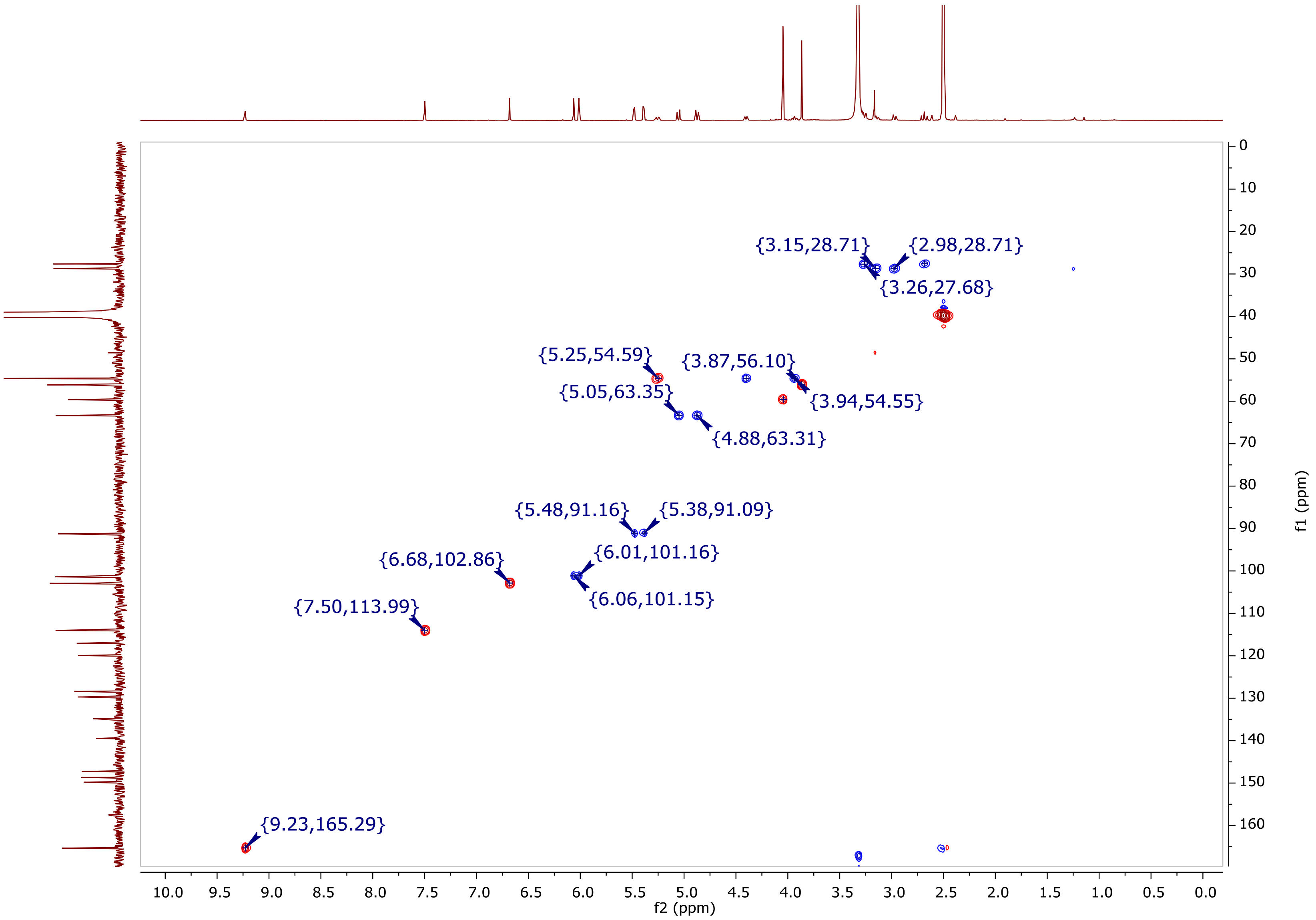

Figure S15. ^{1}H and ^{13}C decoupled HSQC NMR spectrum (600 MHz) of **2** in DMSO-d_6

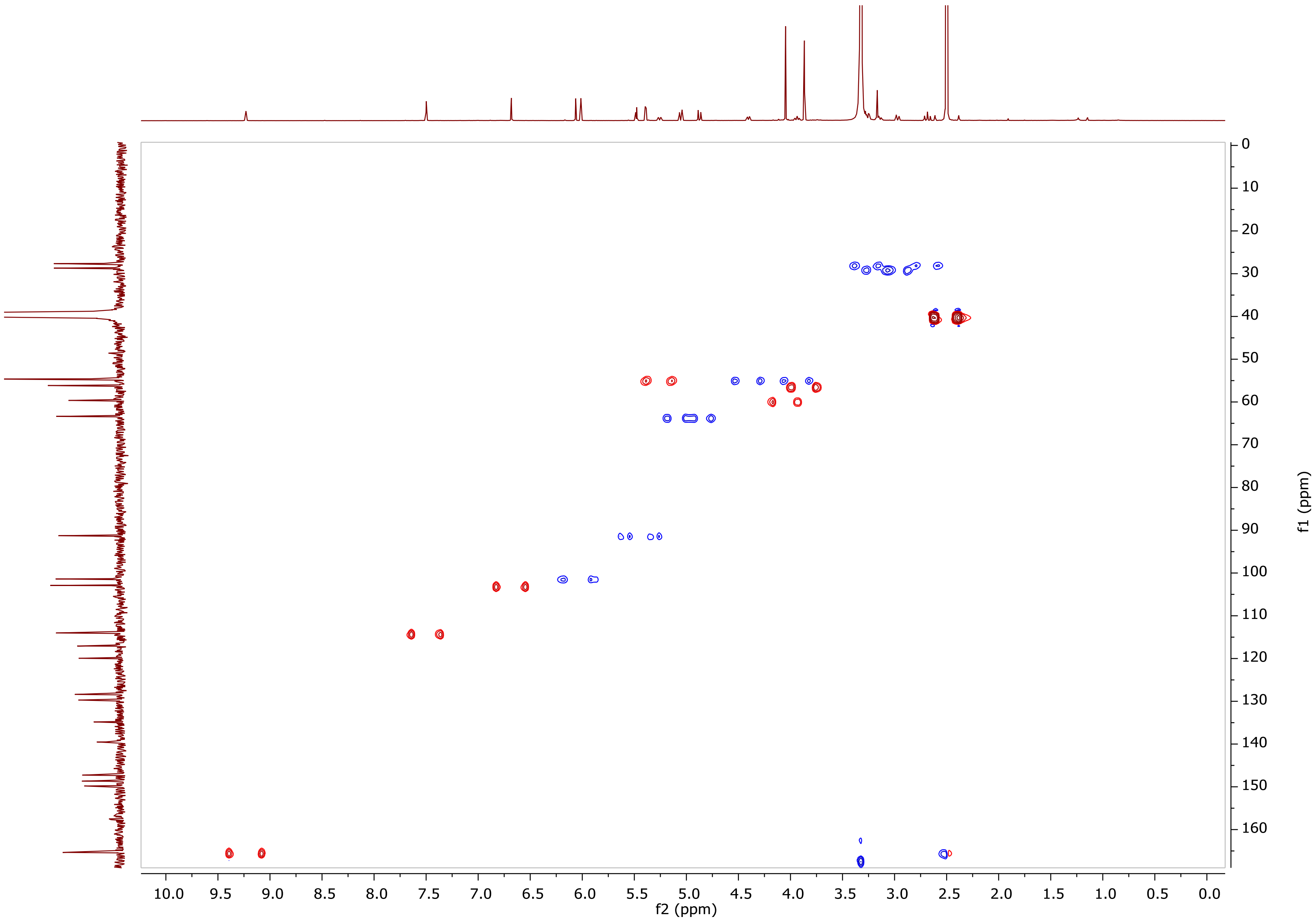

Figure S16. ^{1}H decoupled HSQC NMR spectrum (600 MHz) of **2** in DMSO-d_6

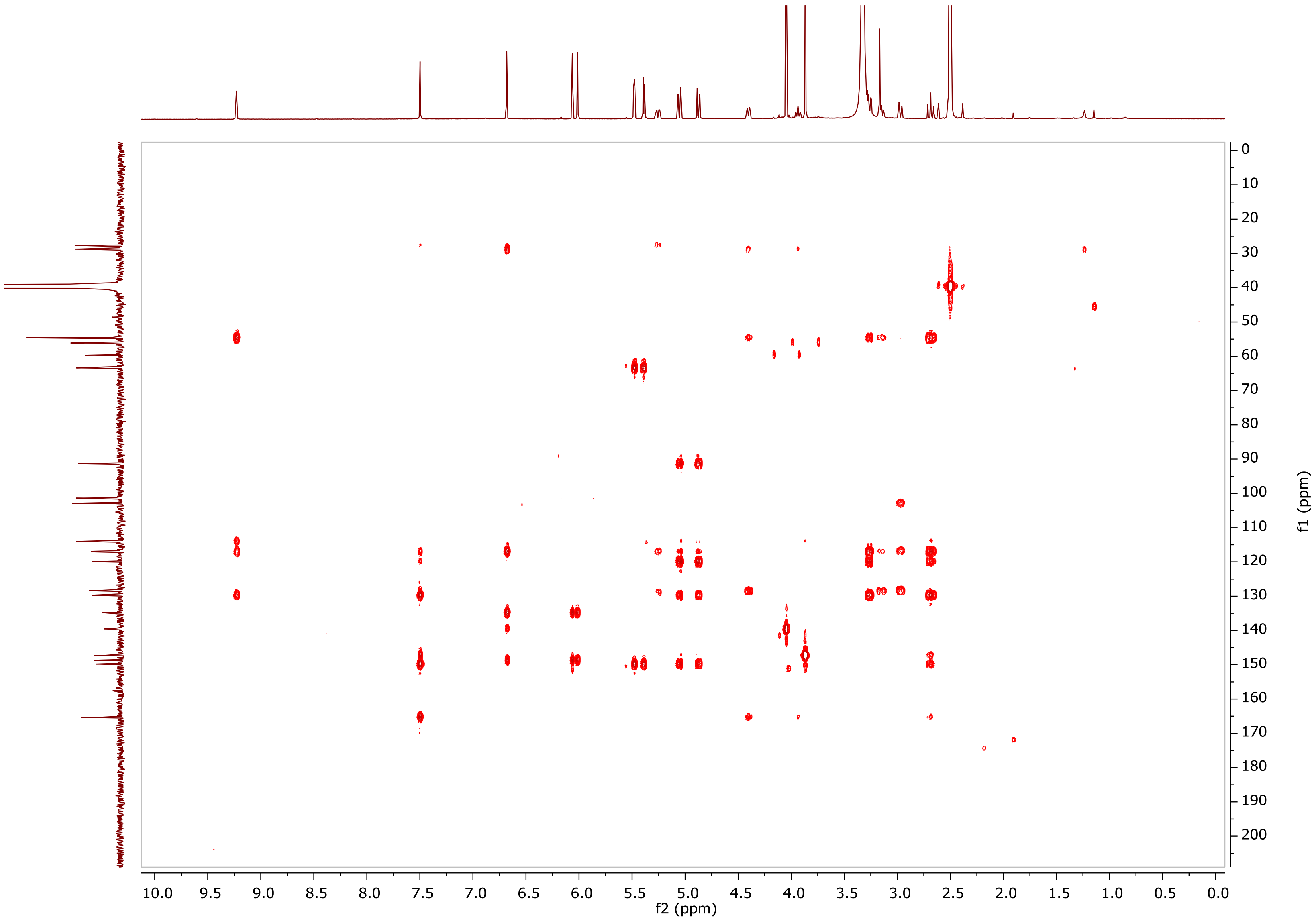

Figure S17. HMBC NMR spectrum (600 MHz) of **2** in DMSO-d_6

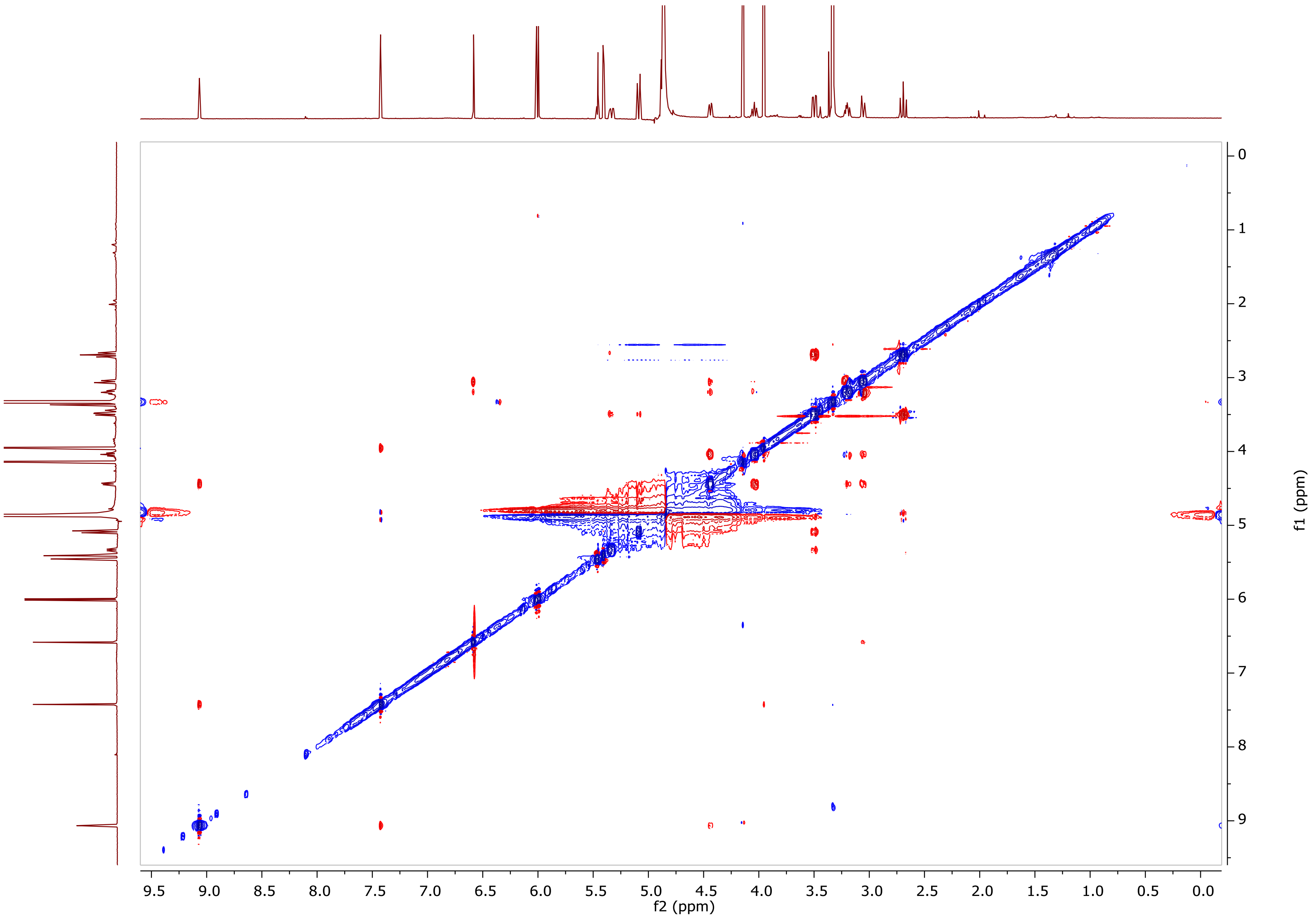

Figure S18. ROESY NMR spectrum (600 MHz) of **2** in DMSO-d_6

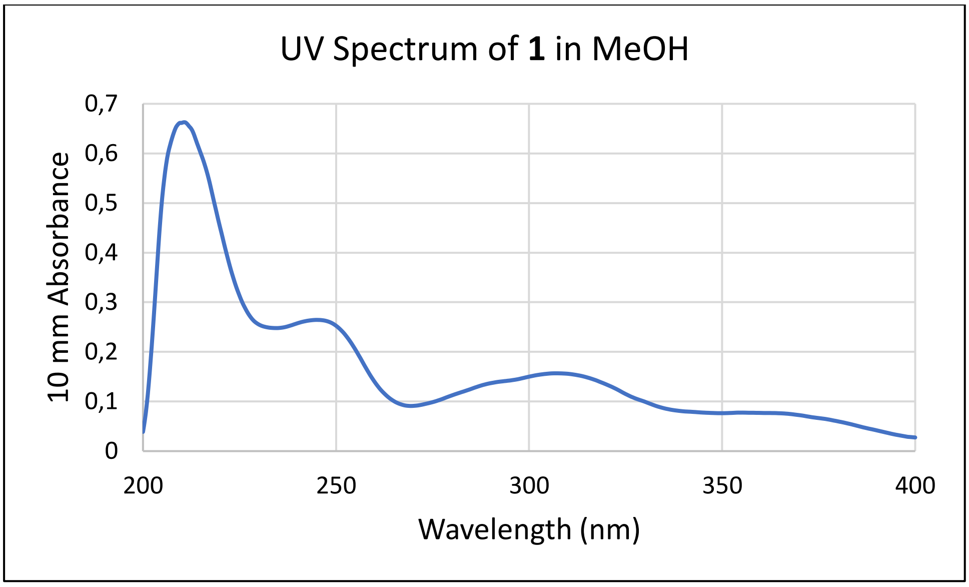

Figure S19. UV spectrum of **1** in MeOH

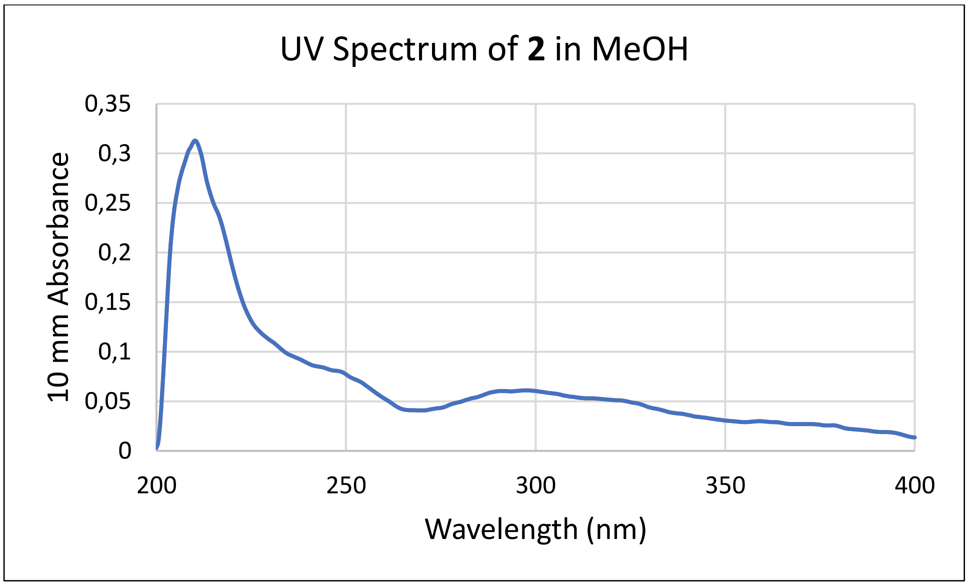

Figure S20. UV spectrum of **2** in MeOH

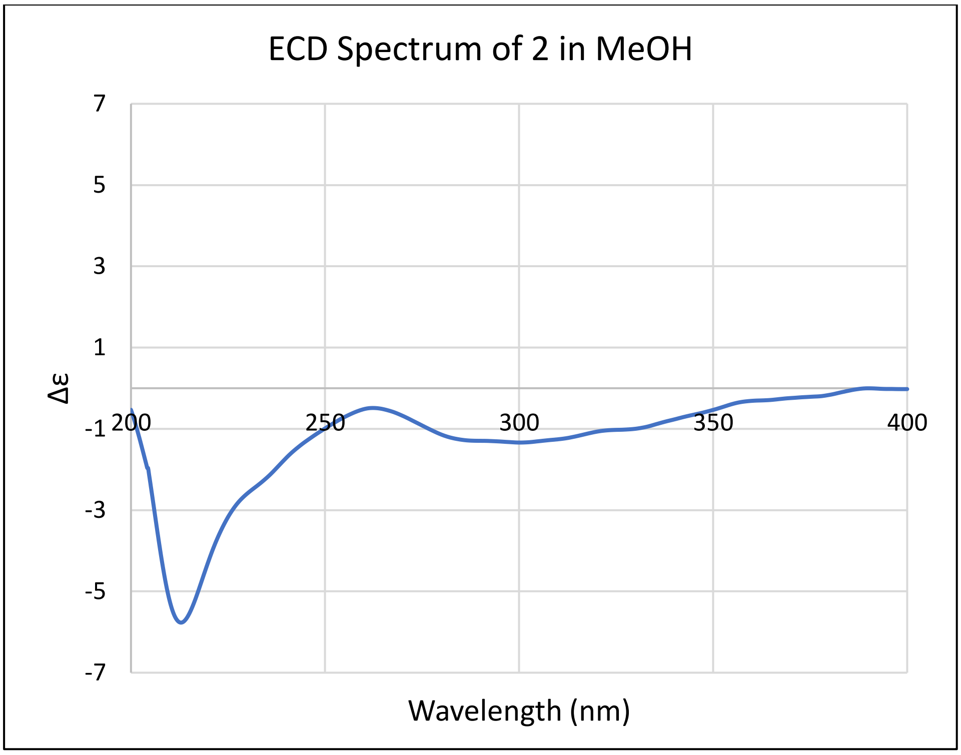

Figure S21. ECD spectrum of **2** in MeOH

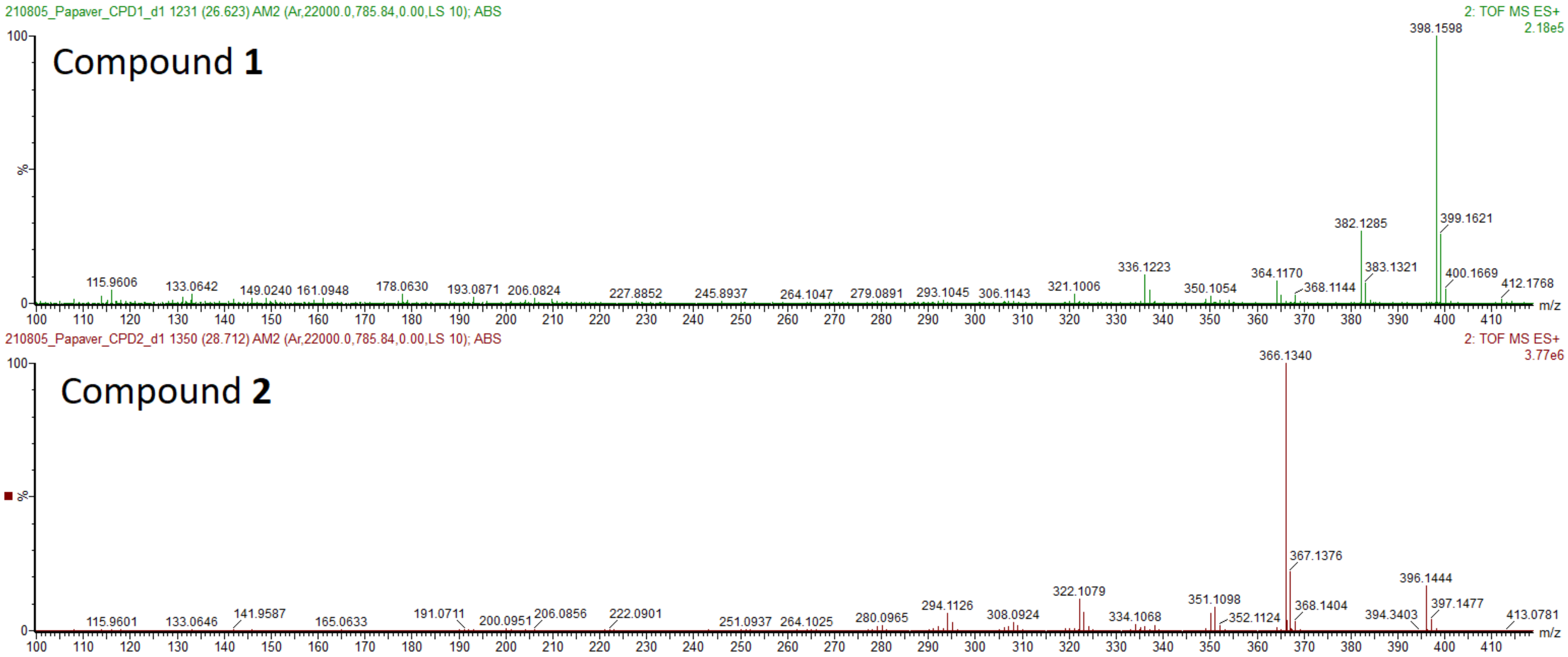

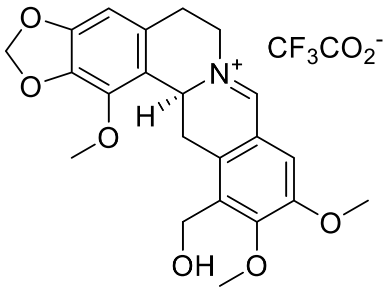

1

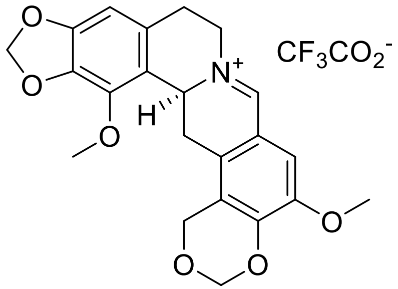

2

Figure S22. MS/MS spectra of **1** and **2**

Table S23. NMR spectroscopic data for 7,8,13,14-dehydroorientalidine (**5**) in methanol-d_4

position	7,8,13,14-dehydroorientalidine (**5**)		
	δ_C[a], type	δ_H[b] (*J* in Hz)	HMBC[c]
1	143.5, C	-	-
2	138.1,[d] C	-	-
3	153.4, C	-	-
4	104.3, CH	6.74, t (0.6)	1, 2, 3, 5, 14a
4a	134.2, C	-	-
5	29.3, CH_2	3.17, m	4, 4a, 6, 14a
6	56.6, CH_2	4.74, m	-
7	N	-	-
8	146.9, CH	9.38, brs	-
8a	123.5, C	-	-
9	106.6, CH	7.59, s	8, 11, 12
10	153.6, C	-	-
11	152.9, C	-	-
12	115.7, C	-	-
12a	133.7, C	-	-
13	118.7, CH	8.62, s	8, 8a, 14, 14a
14	138.0, C	-	-
14a	114.1, C	-	-
15	103.7, CH_2	6.11, d (0.9)	2, 3
16	64.5, CH_2	5.34, s	8a, 9, 11, 12, 12a, 13, 14, 17
MeO-1	60.9, CH_3	4.18, s	1
MeO-10	57.1, CH_3	4.08, s	10
17	93.3, CH_2	5.53, d (0.9)	11, 16

[a]150 Mhz. [b]600 Mhz. [c]HMBC correlations are from proton(s) stated to the indicated carbon. [d]Chemical shift determined from 2D data.

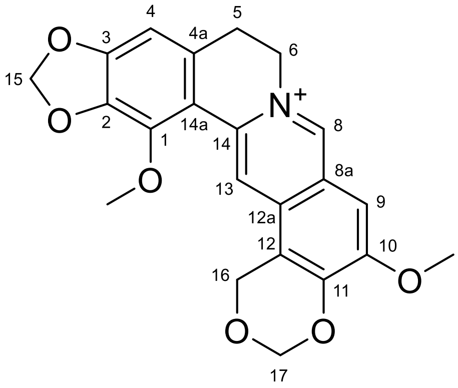

5

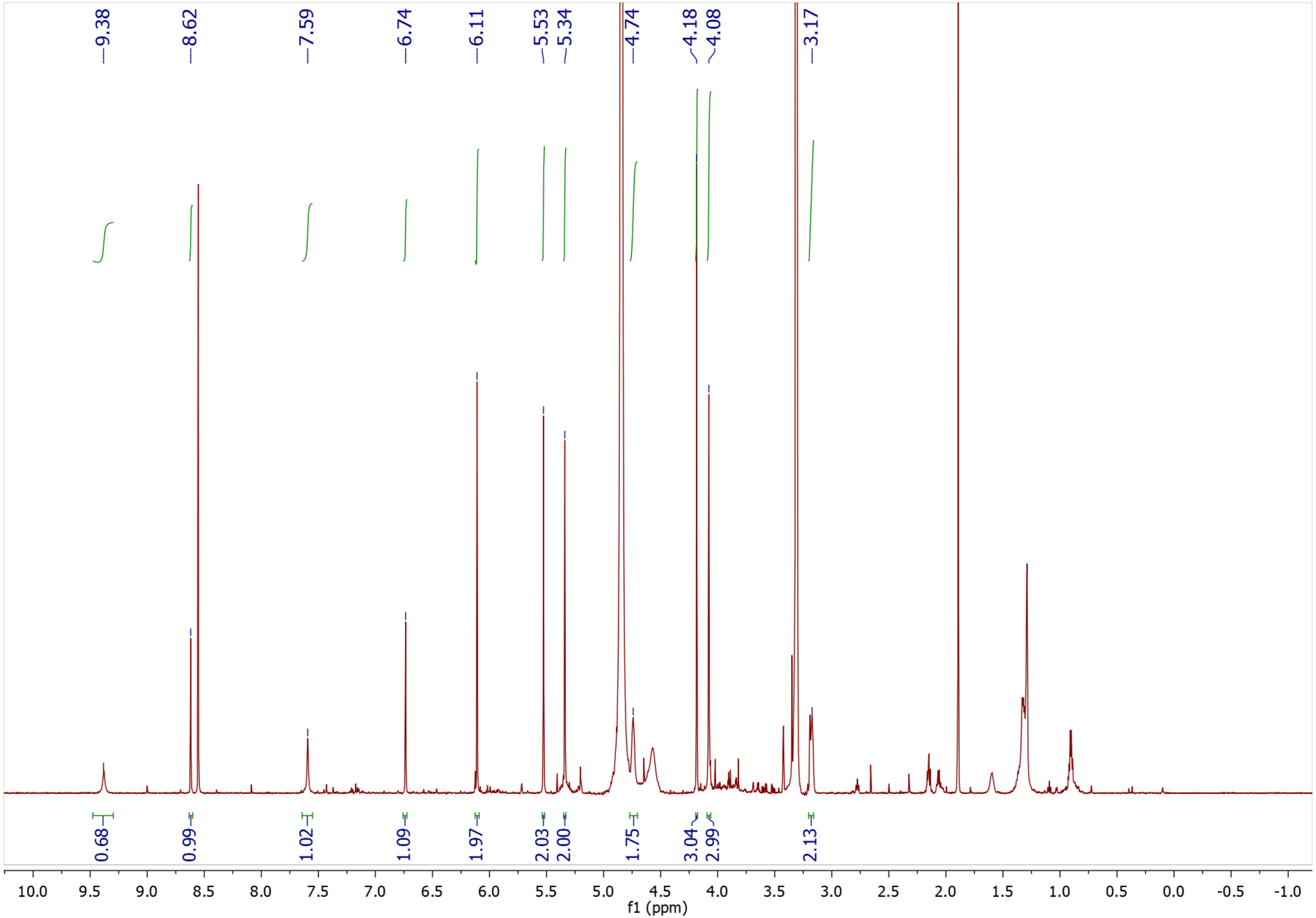

Figure S24. ^{1}H NMR spectrum (600 MHz) of **5** in methanol-d_4

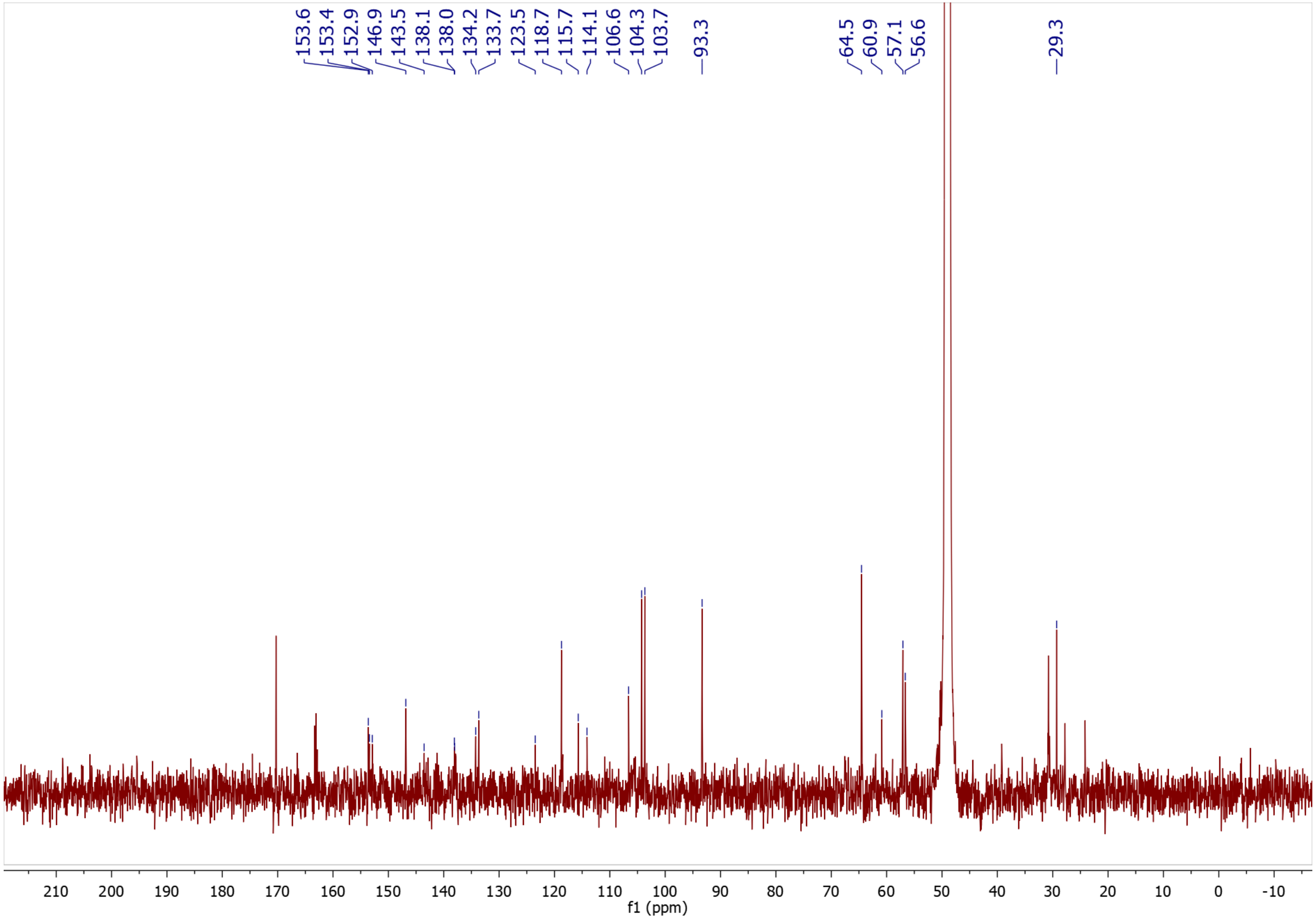

Figure S25. ^{13}C NMR spectrum (150 MHz) of **5** in methanol-d_4

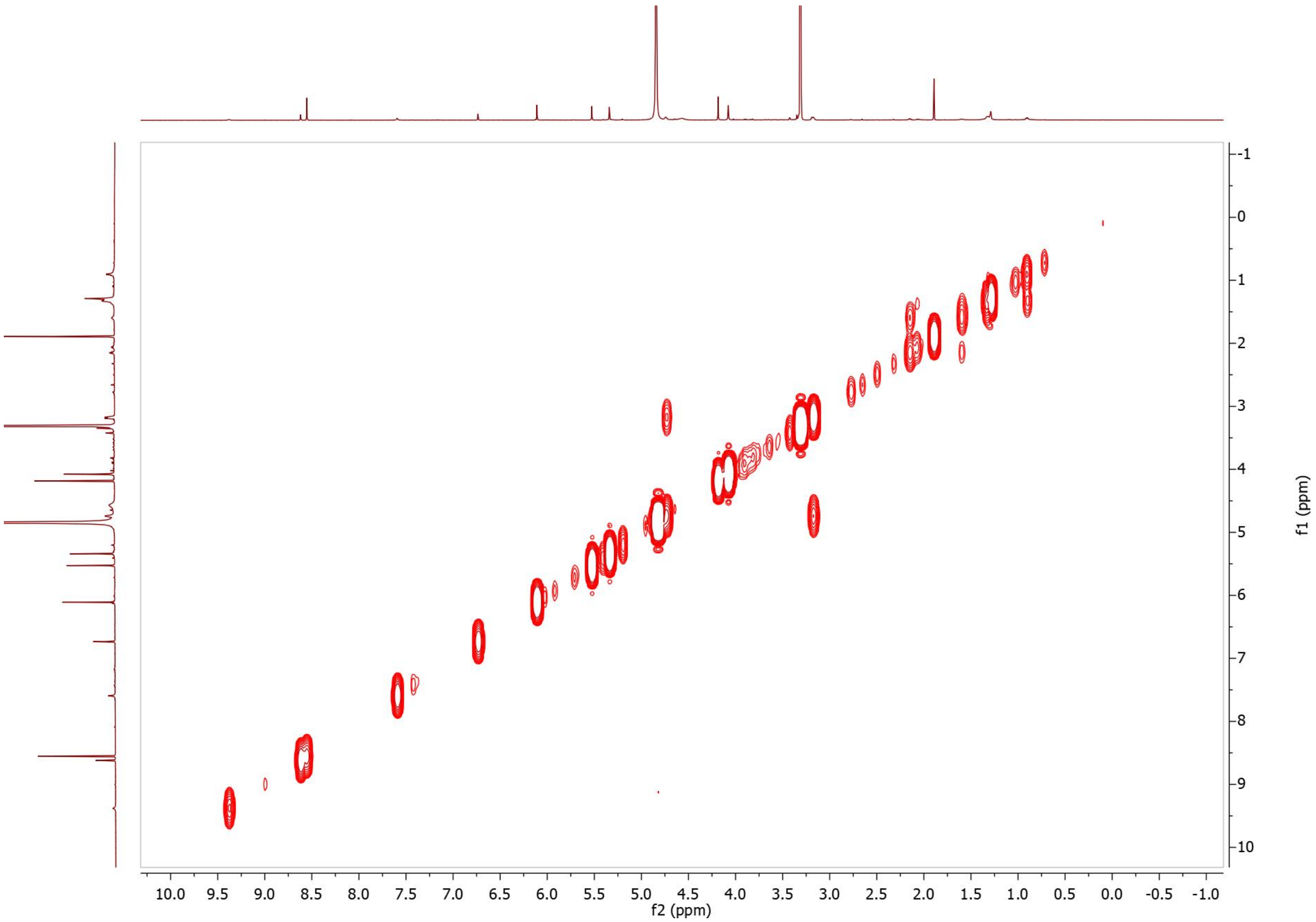

Figure S26. COSY NMR spectrum (600 MHz) of **5** in methanol-d_4

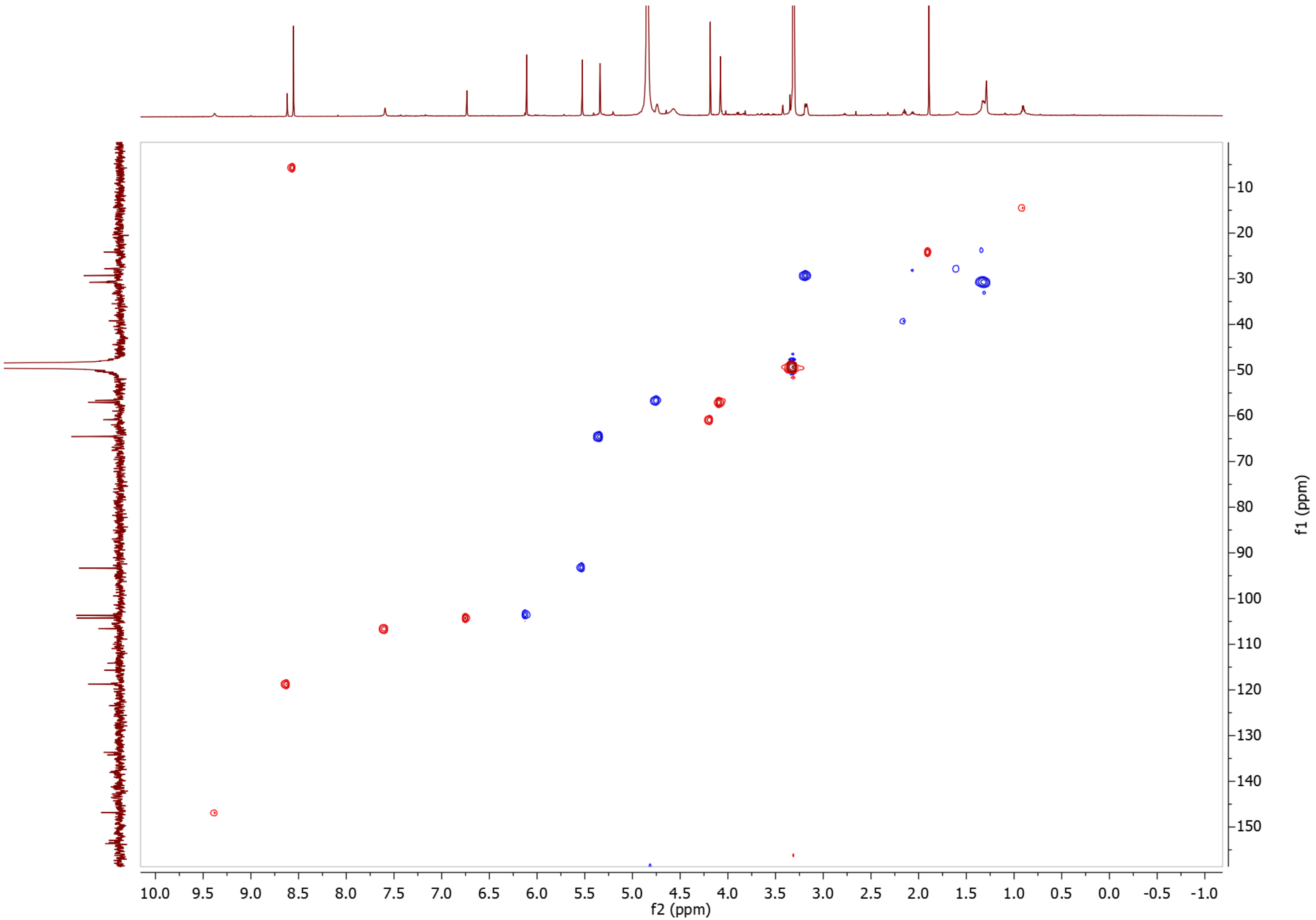

Figure S27. HSQC NMR spectrum (600 MHz) of **5** in methanol-d_4

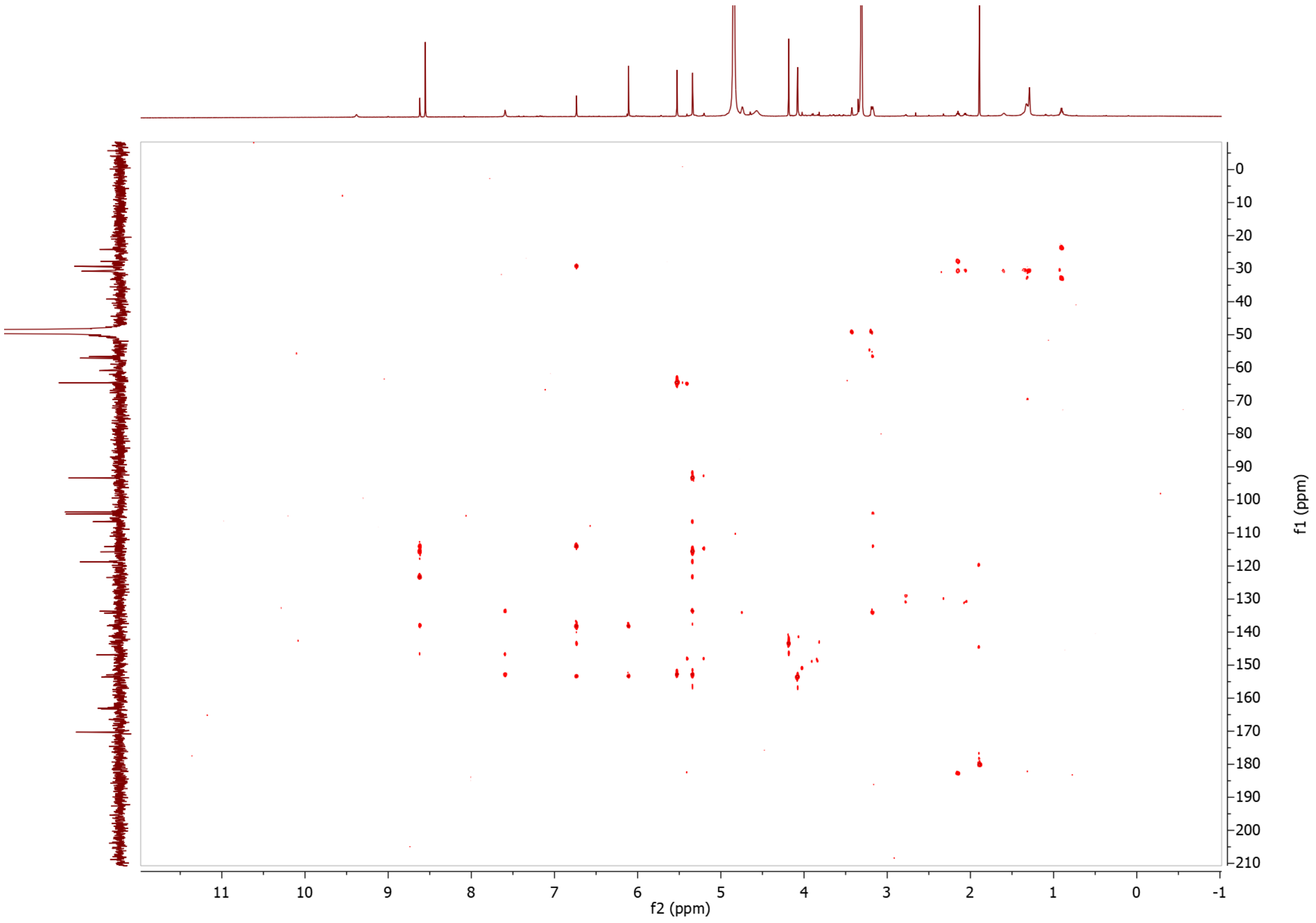

Figure S28. HMBC NMR spectrum (600 MHz) of **5** in methanol-d_4

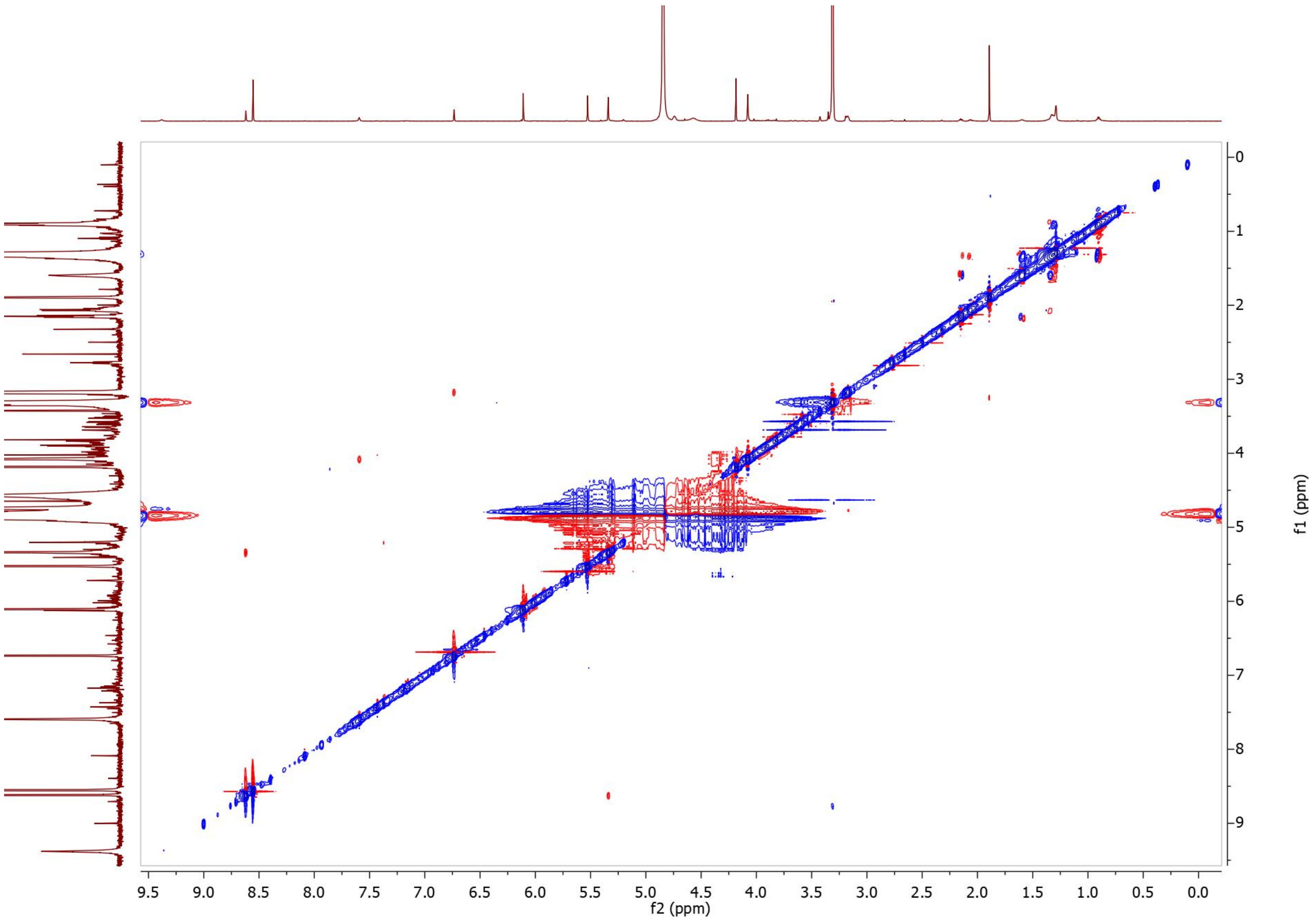

Figure S29. ROESY NMR spectrum (600 MHz) of **5** in methanol-d_4

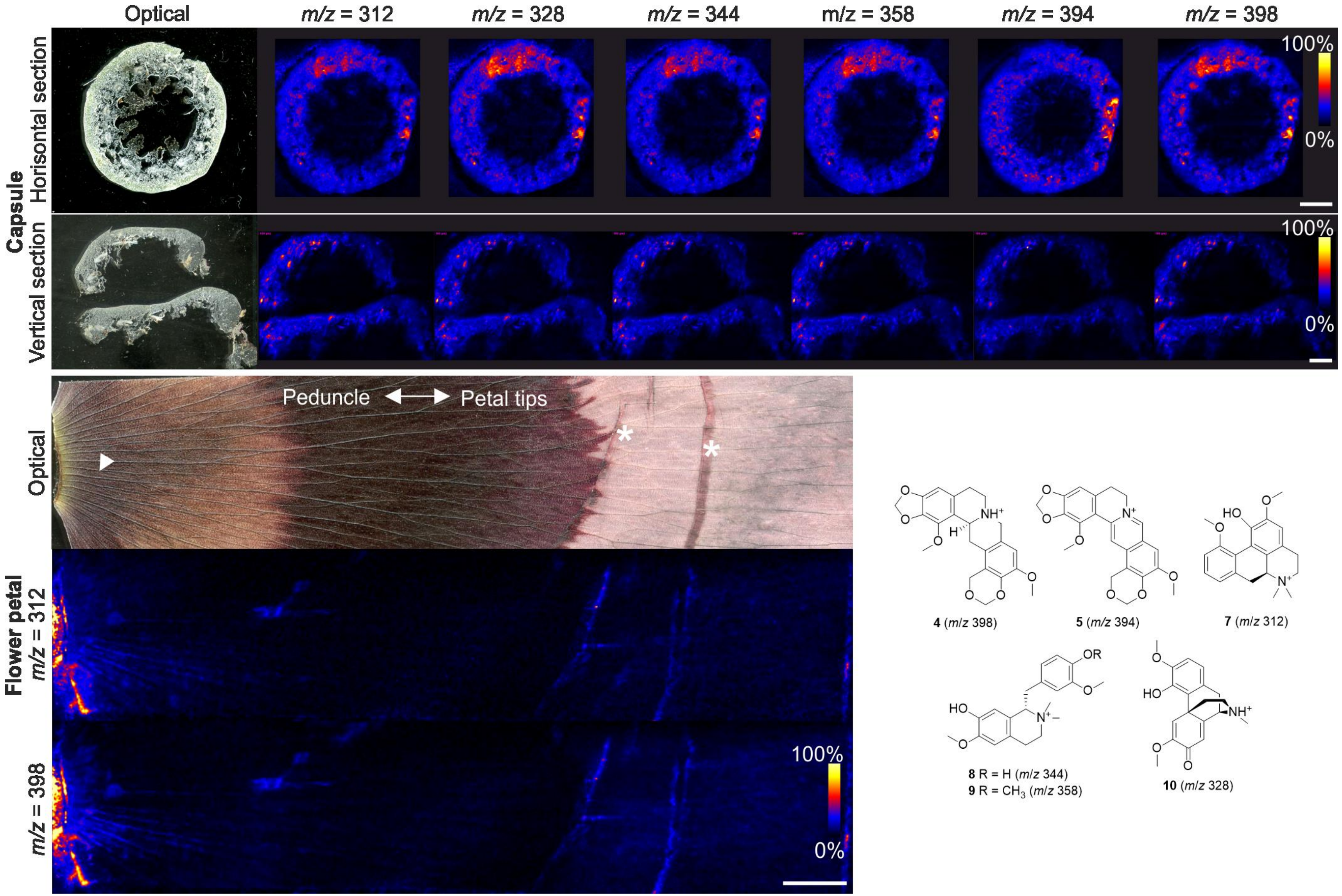

Figure S30. Extra DESI-IMS data of *P. setiferum* capsules. Bar = 3 mm (horizontal section); 3.5 mm (vertical section); 4.5 mm (petals).

Figure S31. Isolation timeline of compounds **1**-**2**.

<u>Monday 27th of July 2020</u>: A whole plant sample of *Papaver pseudo-orientale* is collected from the garden in the morning and placed in an oven (40 °C) at noon after separation of plant into leaves, stems, flowers, and roots.

<u>Tuesday 28th July 2020</u>: Dried flowers are removed from the oven in the morning, ground to a powder with a mortar and pestle (1.6 g) and exhaustively extracted via sonication (45 mins) in MeOH/CH_2Cl_2 (400 mL) and MeOH (800 mL). Both solvents were of analytical grade. The solution was filtered through filter paper under gravity (Whatman Filter Paper 42), then evaporated on a rotary evaporator (40 °C), yielding 0.9 g of extract. The dried extract was stored in an uncapped round bottom flask at room temperature until further use.

<u>Thursday 30th July 2020:</u> The extract (0.9 g) was adsorbed onto C_{18} silica gel (0.9 g) and the extract-impregnated gel was loaded onto a C_{18} flash/MPLC column (Biotage Sfär C_{18}, 12 g, 100 Å, 30 µm). MPLC was performed on Amersham Biosciences ÄKTA FPLC using a P-920 pump, UPC-900 UV detector and Frac-900 fraction collector. The column was then eluted at a flow rate of 10 mL/min with 100% H_2O (0.1% TFA) over five mins, then to 100% CH_3CN (0.1% TFA) over 50 mins. The column was then eluted with 100% CH_3CN (0.1% TFA) for a further five mins. Sixty fractions were collected at one-minute intervals and labelled A.1-A.60. The fractions, which were collected into 15 mL soda lime glass test tubes, were then evaporated overnight on a Genevac HT-4X evaporation system using a 12 hour program, with the temperature not exceeding 40 °C.

<u>Monday 3rd August 2020:</u> Fractions A.23-A.26 (25 mg) from above were redissolved in a small amount of methanol, adsorbed onto C_{18} silica gel (25 mg) and the extract impregnated gel was loaded into an HPLC precolumn cartridge (10 mm x 20 mm) and connected in series to a

C_{18}-bonded silica HPLC column (Kinetex XB-C_{18}, 5 µm, 100 Å, 21.2 x 150 mm). HPLC was performed with a Varian Pro Star pump equipped with a Varian 9050 UV-VIS detector and an LKB 2212 Heurac fraction collector. The extract was purified at a flow rate of 9 mL/min using a gradient from 95% H_2O (0.1% TFA)/5% CH_3CN (0.1% TFA) to 85% H_2O (0.1% TFA)/15% CH_3CN (0.1% TFA) over 5 mins, then to 65% H_2O (0.1% TFA)/35% CH_3CN (0.1% TFA) over 90 mins. Ninety-five fractions were collected and labelled B.1-B.95. The fractions, which were collected into 15 mL soda lime glass test tubes, were then evaporated overnight on a Genevac HT-4X evaporation system using a 12 hour program, with the temperature not exceeding 40 °C.

Tuesday 4th August 2020: The samples were directly redissolved in methanol-d_4 or DMSO-d_6 in the morning and analysed with ^{1}H NMR in the afternoon.

Wednesday 5th August 2020: Two-dimensional NMR data is acquired on **2** (DMSO-d_6) and **1** (methanol-d_4) in the afternoon and overnight.

Monday 10th August 2020: Compound **1** (dissolved in methanol-d_4) is transferred out of its NMR tube, evaporated on a Genevac HT-4X evaporation system in a 2 ml glass vial using a two hour program, with the temperature not exceeding 40 °C. The sample is dissolved in DMSO-d_6 and two-dimensional NMR data is acquired the same evening. No compound degradation was observed.

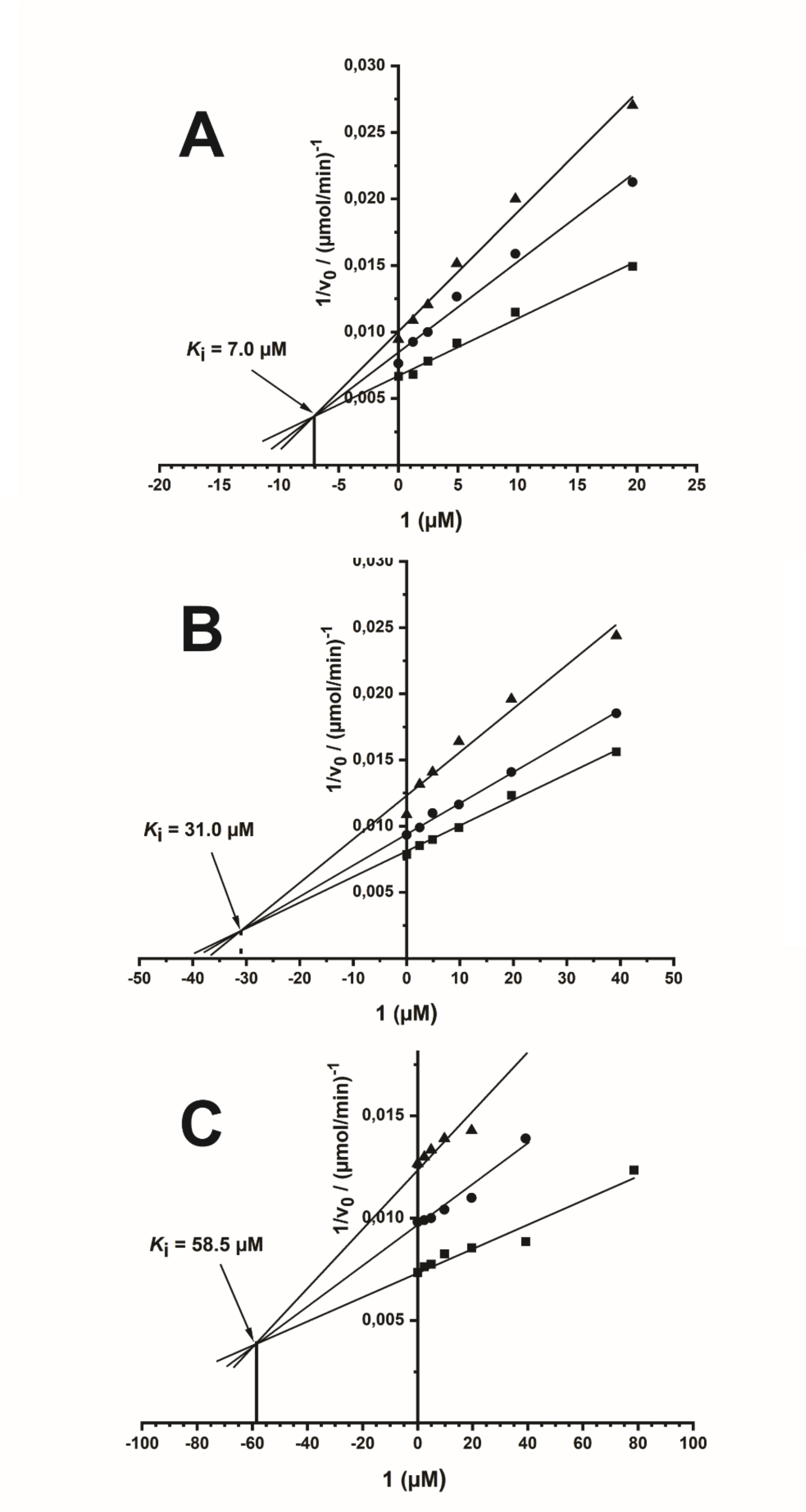

Figure S32. Dixon plots for determination of type of inhibition and inhibition constants (K_i) for compound **1** against electric eel acetylcholinesterase (**A**), human recombinant acetylcholinesterase (**B**) and horse serum butyrylcholinesterase (**C**). Substrate concentrations: 0.125 mM (▲), 0.25 mM (●), 0.5 mM (■).

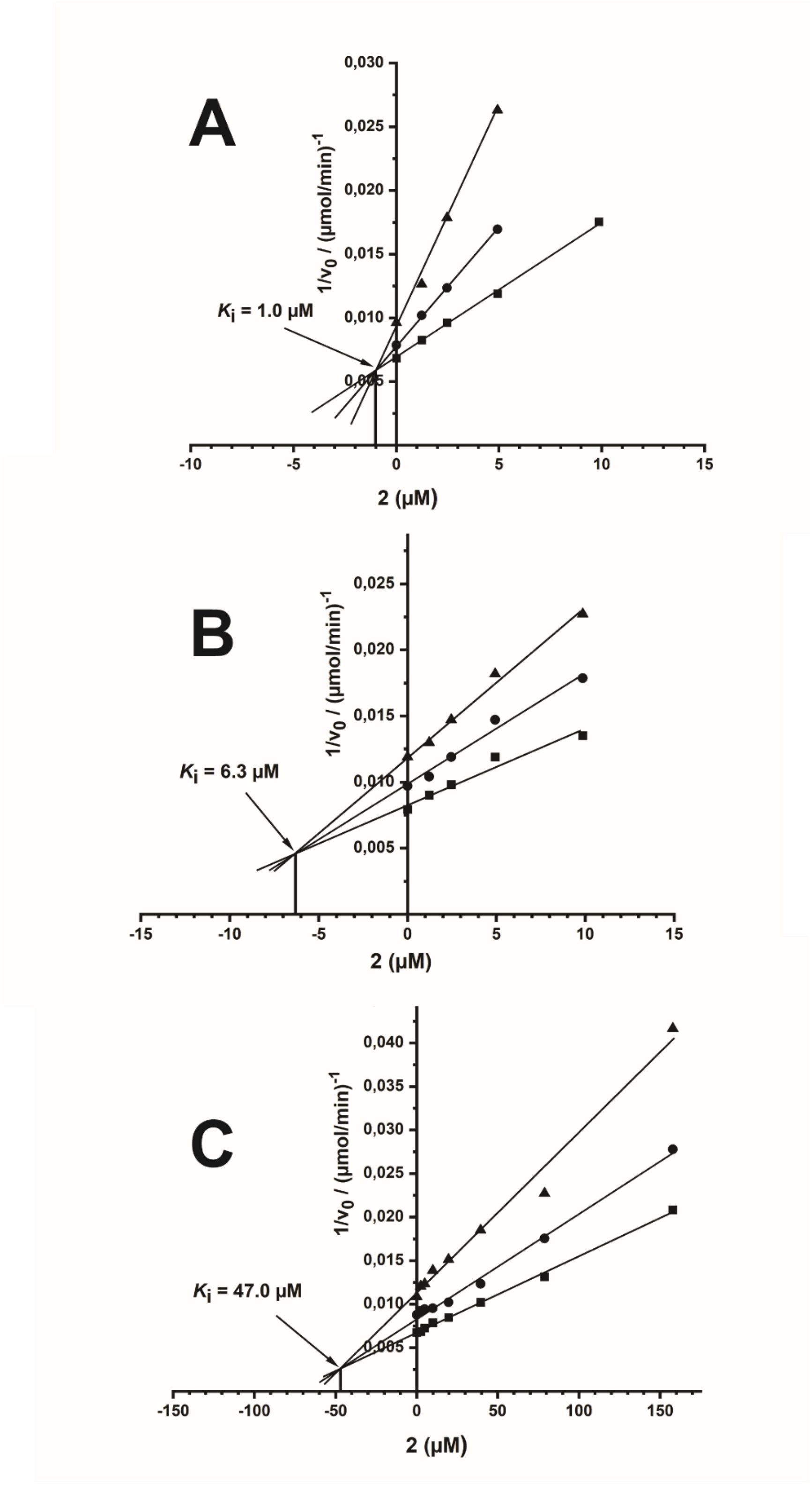

Figure S33. Dixon plots for determination of type of inhibition and inhibition constants (K_i) for compound **2** against electric eel acetylcholinesterase (**A**), human recombinant acetylcholinesterase (**B**) and horse serum butyrylcholinesterase (**C**). Substrate concentrations: 0.125 mM (▲), 0.25 mM (●), 0.5 mM (■).

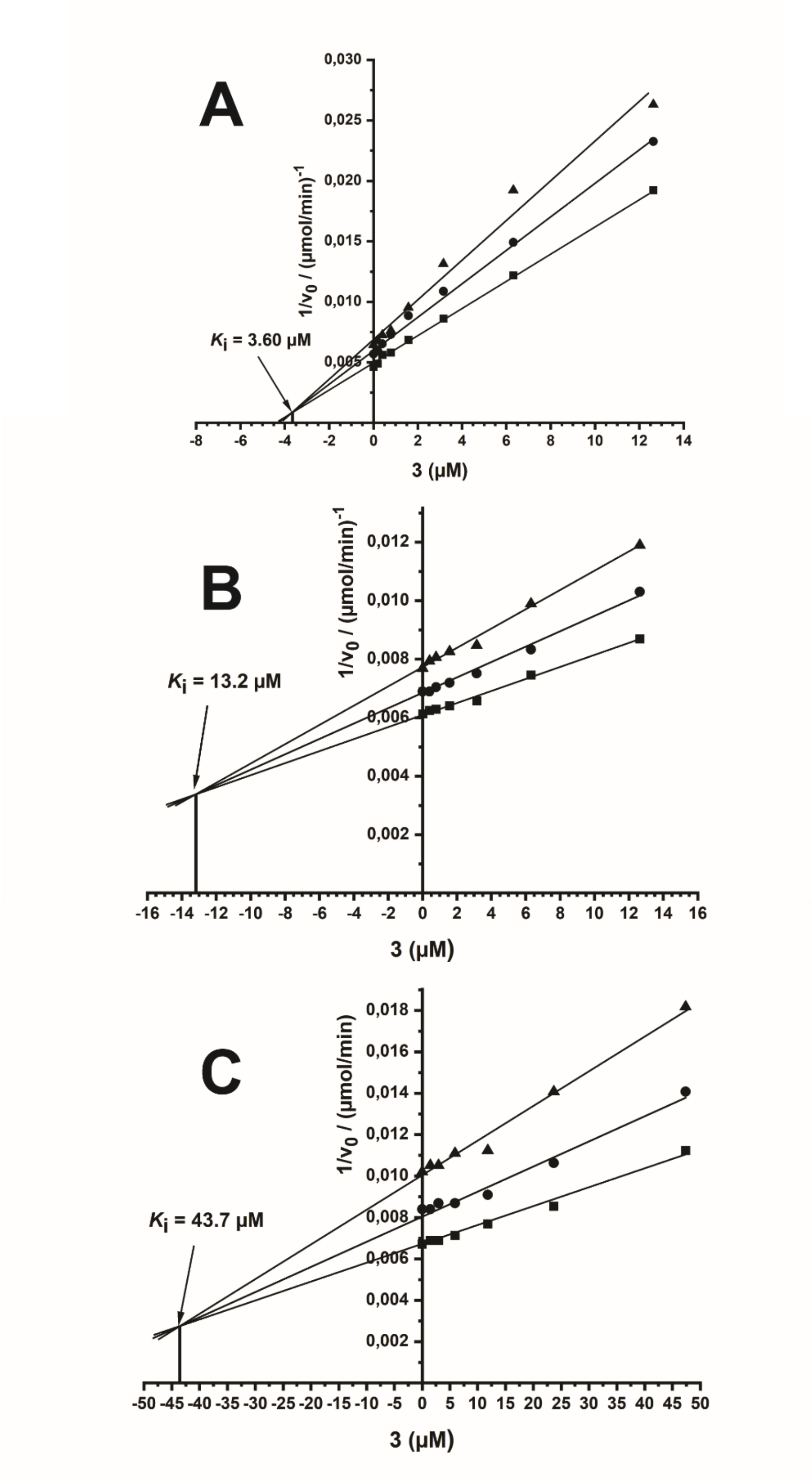

Figure S34. Dixon plots for determination of type of inhibition and inhibition constants (K_i) for compound **3** against electric eel acetylcholinesterase (**A**), human recombinant acetylcholinesterase (**B**) and horse serum butyrylcholinesterase (**C**). Substrate concentrations: 0.125 mM (▲), 0.25 mM (●), 0.5 mM (■).

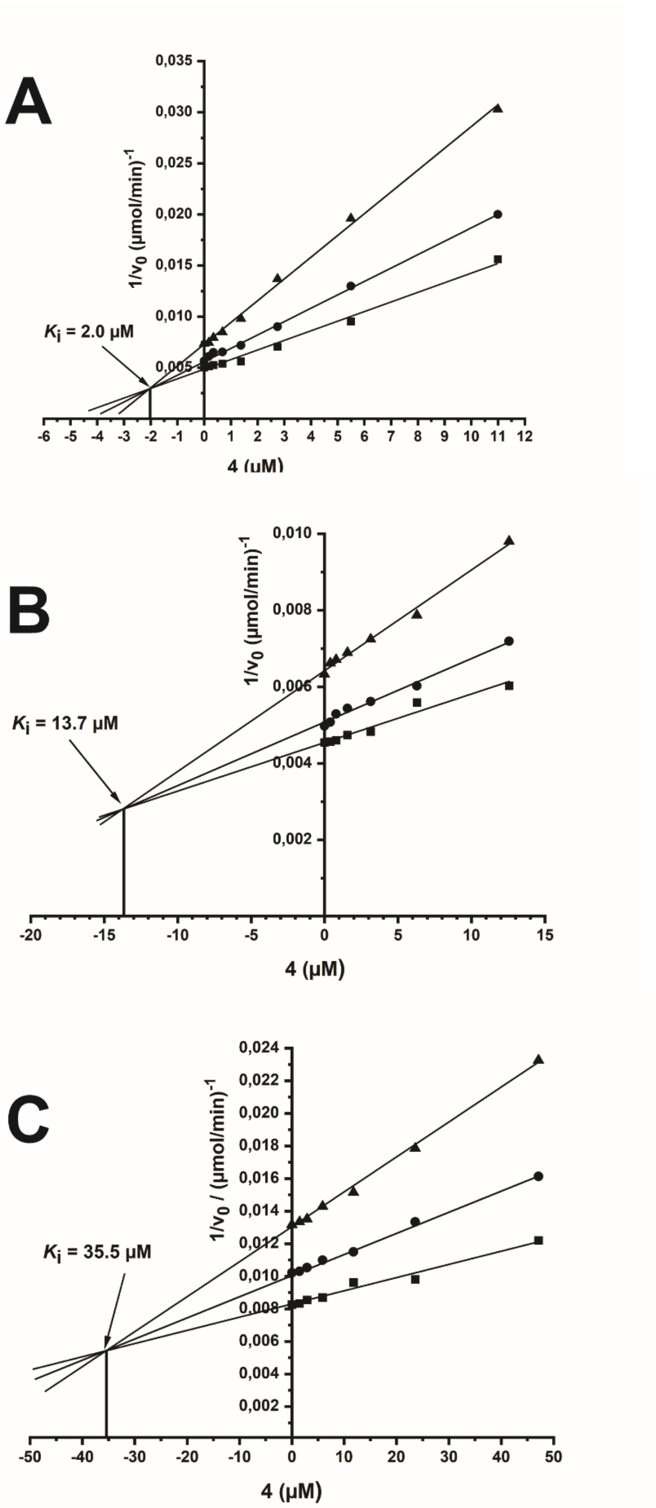

Figure S35. Dixon plots for determination of type of inhibition and inhibition constants (K_i) for compound **4** against electric eel acetylcholinesterase (**A**), human recombinant acetylcholinesterase (**B**) and horse serum butyrylcholinesterase (**C**). Substrate concentrations: 0.125 mM (▲), 0.25 mM (●), 0.5 mM (■).

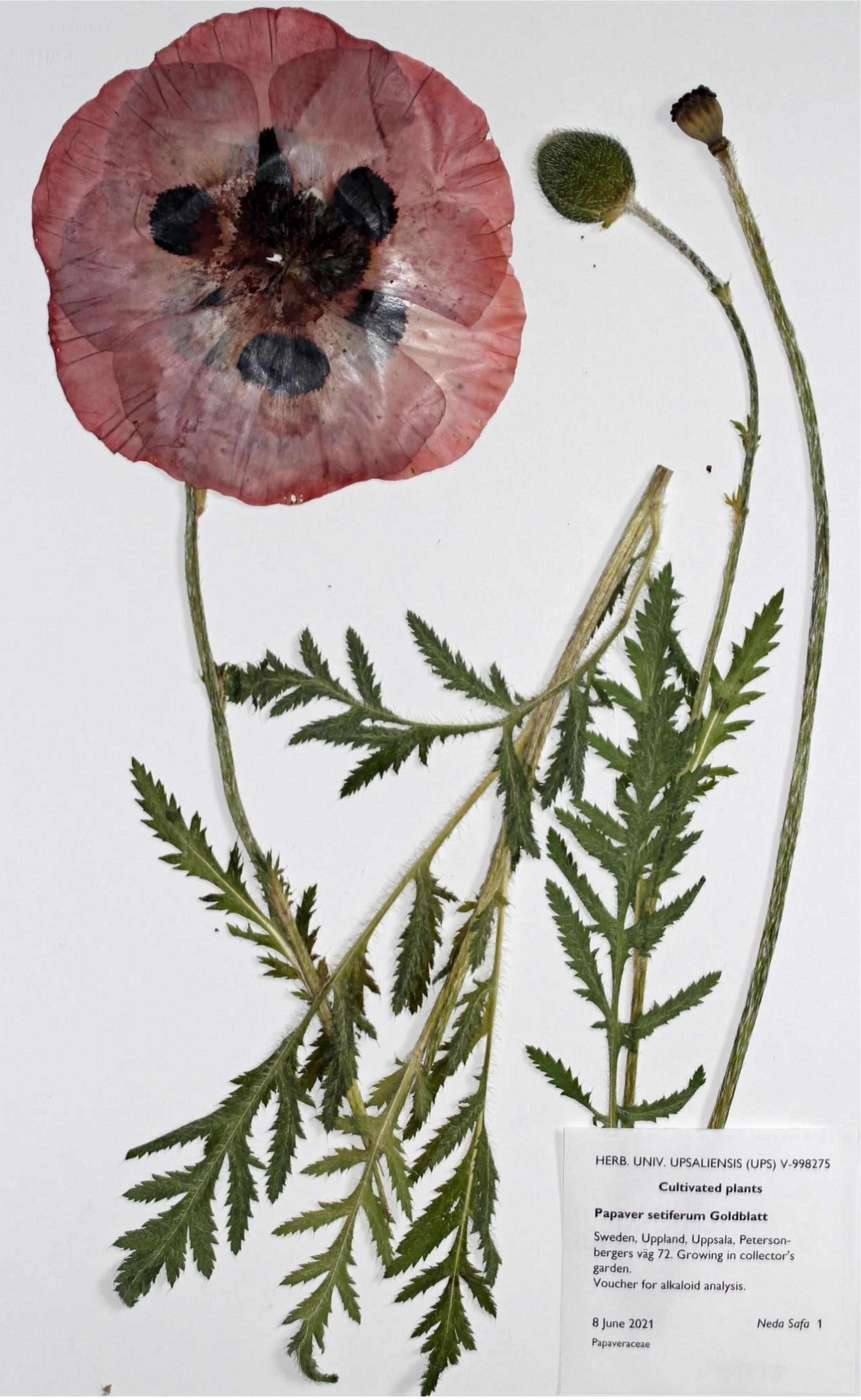

Figure S36. Voucher specimen of *Papaver setiferum*.

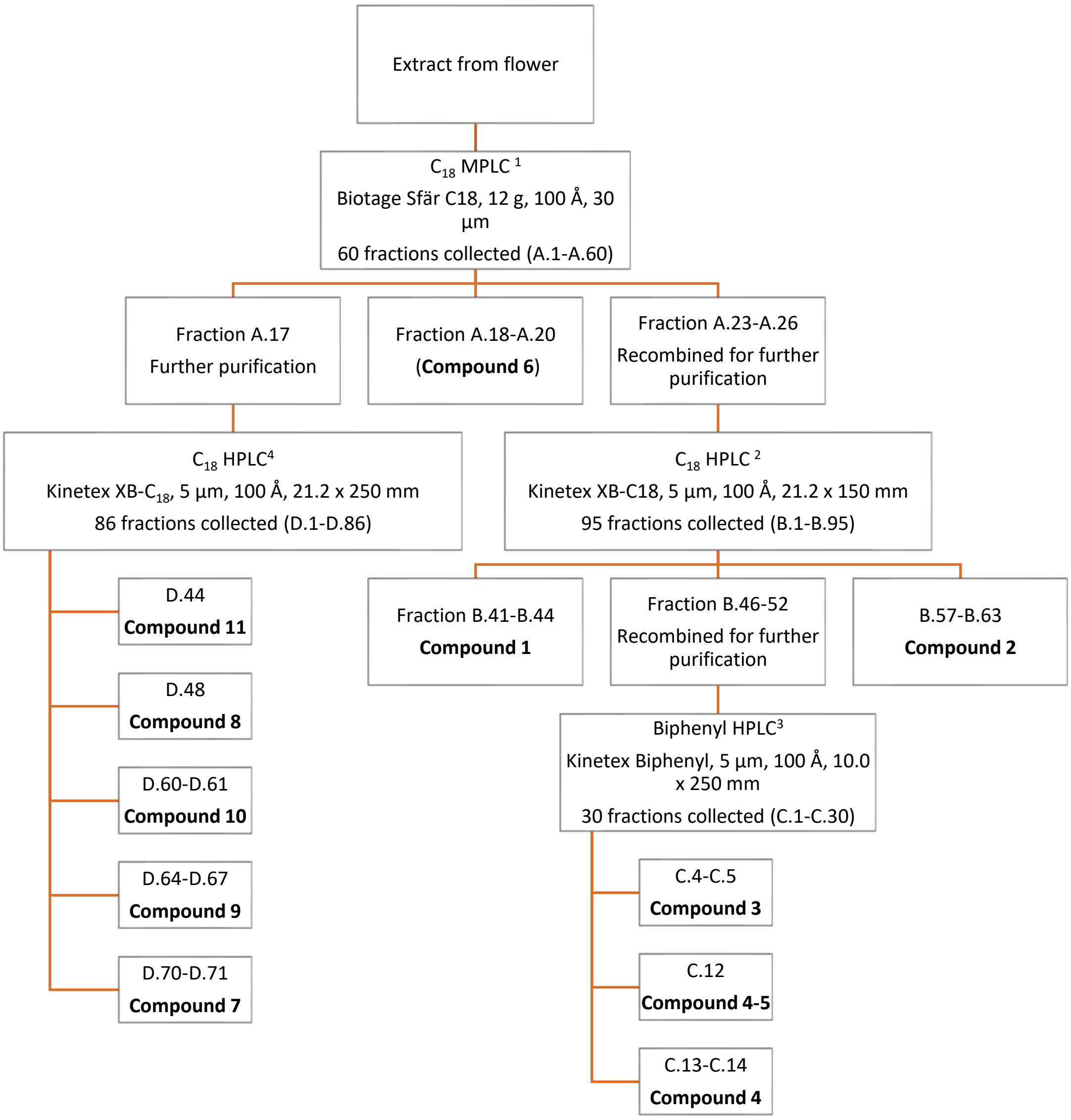

1. MPLC gradient: 100% H_2O (0.1% TFA) over five mins, then to 100% CH_3CN (0.1% TFA) over 50 mins. The column was then eluted with 100% CH_3CN (0.1% TFA) for a further five mins. Flow rate: 10 mL/min.
2. HPLC gradient: From 95% H_2O (0.1% TFA)/5% CH_3CN (0.1% TFA) to 85% H_2O (0.1% TFA)/15% CH_3CN (0.1% TFA) over 5 mins, then to 65% H_2O (0.1% TFA)/35% CH_3CN (0.1% TFA) over 90 mins. Flow rate: 9 mL/min.
3. HPLC gradient: From 95% H_2O (5 mM NH_4OAc, pH 4.8)/5% CH_3CN to 62% H_2O (5 mM NH_4OAc, pH 4.8)/38% CH_3CN over five mins, then to 58% H_2O (5 mM NH_4OAc, pH 4.8)/42% CH_3CN over 20 mins, then to 5% H_2O (5 mM NH_4OAc, pH 4.8)/95% CH_3CN over 5 mins. Flow rate: 4mL/min.
4. From 95% H_2O (0.1% TFA)/5% CH_3CN (0.1% TFA) to 80% H_2O (0.1% TFA)/20% CH_3CN (0.1% TFA) over 76 mins, then to 5% H_2O (0.1% TFA)/95% CH_3CN (0.1% TFA) over 10 mins. Flow rate: 9 mL/min.

Figure S37. Isolation flow chart of compounds **1-11**.
